# Ketal Protection of Glycerol for Selective Electrosynthesis of Glyceric Acid in Highly Alkaline Media

**DOI:** 10.1002/anie.5555743

**Published:** 2026-06-18

**Authors:** Jiamin Wang, Baokun Zhang, Kanglei Pang, Egon Campos dos Santos, Hong Liu, Mats Johnsson, Xiaowen Yu

**Affiliations:** ^1^ State Key Laboratory of Crystal Materials Shandong University Jinan People's Republic of China; ^2^ Department of Chemistry Stockholm University Stockholm Sweden; ^3^ Departamento De Ciências Naturais Universidade Federal de São João Del‐Rei São João del‐Rei Minas Gerais Brazil

**Keywords:** electrocatalysis, glyceric acid, glycerol oxidation, ketal protection, solketal oxidation

## Abstract

Electrocatalytic glycerol oxidation represents a sustainable route for glycerol valorization, yet high selectivity toward glyceric acid (GLA) remains challenging on non‐noble‐metal catalysts due to competing C─C bond cleavage and complex oxidation pathways. Herein, we introduce solketal, a ketal‐protected glycerol derivative, to steer reaction selectivity. Its rigid five‐membered ketal structure selectively exposes the primary hydroxyl group while shielding vicinal diols, thereby enforcing site‐selective oxidation and suppressing C–C scission. Using a non‐noble Cu_3_Mo_2_O_9_ catalyst in 6.0 M KOH with 0.2 M solketal, the system achieves a record‐high GLA selectivity of 90.1% with a production rate of 675.45 µmol cm^−2^ h^−1^ at 1.40 V versus RHE, reaching ∼95% substrate conversion within 5 h and maintaining stable operation over 100 h. Mechanistic investigations reveal that highly alkaline conditions promote solketal deprotonation to reactive alkoxide species, weaken the C_α_─H bond to accelerate dehydrogenation kinetics, and mitigate local interfacial acidification, thus stabilizing the ketal‐protected intermediate and suppressing side reactions. Furthermore, practical feasibility is demonstrated through integrated upstream solketal synthesis (90% yield, 99% purity) and downstream GLA isolation (99% purity). This work establishes a substrate‐protection strategy for steering reaction pathways, offering new insights into the selective conversion of biomass‐derived polyols into value‐added oxygenates.

## Graphical abstract

Using a ketal‐protected glycerol derivative (solketal) as the oxidation substrate enables site‐selective electrooxidation that suppresses C–C cleavage on non‐noble catalysts. Highly alkaline conditions promote reactive solketal alkoxide formation, weaken the C_α_─H bond to facilitate dehydrogenation, and buffer local acidification, enabling a record‐high glyceric acid selectivity of 90.1% over a Cu_3_Mo_2_O_9_ catalyst.

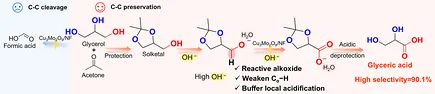

## 1 Introduction

Continued consumption of fossil fuels and the associated environmental challenges accelerate the global transition toward green and sustainable energy systems [1, 2]. In this context, biodiesel has emerged as an important renewable alternative to petroleum‐derived diesel, and its industrial production continues to expand [3, 4, 5]. However, biodiesel manufacturing generates large quantities of crude glycerol as a low‐value by‐product—approximately 1 ton of glycerol is produced per 10 tons of biodiesel [6, 7]. This oversupply has depressed glycerol prices to ∼$0.17 kg^−1^, creating both economic and environmental challenges [8, 9]. Currently, the disposal of excess glycerol still relies heavily on conventional methods such as incineration or landfill, which not only wastes valuable carbon resources but also imposes additional environmental burdens [10]. Developing efficient routes to upgrade glycerol into value‐added chemicals is therefore essential for improving biomass utilization and advancing carbon recycling and emission‐reduction goals.

Electrocatalytic glycerol oxidation reaction (GOR) has attracted growing interest as a green and energy‐efficient glycerol upgrading strategy, owing to its mild operating conditions, compatibility with renewable electricity, and potential integration with the hydrogen evolution reaction (HER) [11, 12]. In such paired electrolysis systems, glycerol is converted to value‐added oxygenates at the anode while H_2_ is simultaneously produced at the cathode [13, 14]. Glycerol contains three oxidizable hydroxyl groups, allowing its electrooxidation to yield a wide range of high‐value products, including dihydroxyacetone (DHA), glyceraldehyde (GLAD), glyceric acid (GLA), oxalic acid (OA), glycolic acid (GA), acetic acid (AA), and formic acid (FA) [15, 16, 17]. Among these, GLA is a particularly attractive C_3_ platform molecule with broad application prospects in pharmaceuticals, cosmetics, and biodegradable polymer synthesis [18, 19]. Notably, its market value ($7.5 kg^−1^) significantly exceeds that of C_1_ products such as FA ($0.48 kg^−1^) [20, 21]. Despite this promise, achieving high selectivity toward GLA remains challenging because glycerol electrooxidation proceeds through complex reaction networks and is often accompanied by competing C─C bond cleavage.

**SCHEME 1:**
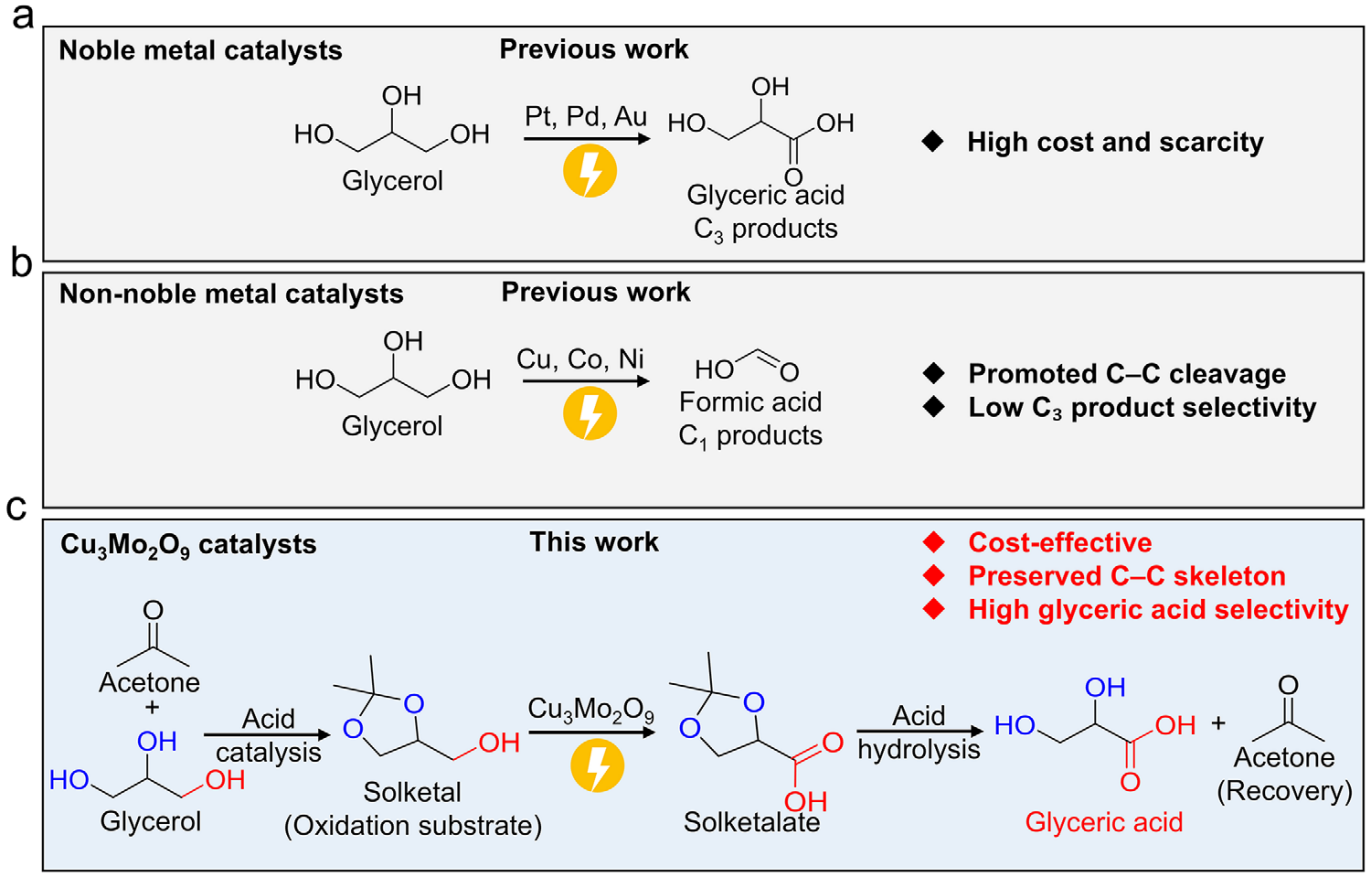
Comparison of GOR pathways reported previously and in this work. (a) Selective oxidation of glycerol to GLA on noble‐metal catalysts. (b) Oxidation of glycerol to FA *via* C−C cleavage on non‐noble metal catalysts. (c) This work: highly selective oxidation of glycerol to GLA enabled by a ketal protection strategy using a Cu_3_Mo_2_O_9_ non‐noble metal electrocatalyst under highly alkaline conditions.

Currently, most GOR studies are conducted in alkaline electrolytes, where hydroxide ions (OH^−^) effectively promote glycerol deprotonation and accelerate reaction kinetics [22, 23]. Under such conditions, noble‐metal catalysts (e.g., Au, Pt, Pd, and their alloys) exhibit outstanding performance, enabling the selective conversion of glycerol into value‐added C_3_ products such as GLA at low potentials (Scheme 1a) [18, 24, 25]. For instance, Wang et al. reported a Pt‐based nanocatalyst comprising a Pt‐Cu‐rich core and a high‐entropy alloy surface, which delivered a high GLA selectivity of 75.2% [18]. Nevertheless, the scarcity and high cost of noble metals severely limit their practical scalability [26]. In contrast, earth‐abundant transition‐metal catalysts (e.g., Cu, Co, and Ni) offer cost‐effective alternatives, yet they generally favor C─C bond cleavage, leading predominantly to the production of low‐value C_1_ products such as FA (Scheme 1b) [27, 28, 29]. Although approaches such as introducing borate or carbonate buffers under weakly alkaline conditions (pH 8.5–10.0) have been explored to suppress C–C scission and steer selectivity toward C_3_ products (e.g., DHA, GLAD) on non‐noble metals, these systems often suffer from low current densities (< 20 mA cm^−2^) and limited control over single‐product distribution [30, 31, 32]. Therefore, developing rational strategies that simultaneously deliver high activity and high GLA selectivity on non‐noble‐metal‐based catalysts remains a critical challenge.

To address this limitation, we strategically replaced glycerol with its ketal‐protected derivative, solketal (isopropylidene glycerol), as an alternative oxidation substrate (Scheme 1c). Solketal can be readily synthesized *via* acid‐catalyzed acetalization of glycerol with acetone [33, 34]. Its acetal protecting group is stable under alkaline conditions yet can be readily hydrolyzed in acidic media [35], making solketal compatible with alkaline GOR environments and enabling facile post‐reaction deprotection. Notably, the rigid five‐membered ring of solketal provides strong steric hindrance that effectively shields the vicinal C_2_ and C_3_ hydroxyl groups, while leaving the primary hydroxyl group at the C_1_ position accessible. This substrate‐level protection is expected to enforce site‐selective oxidation at the primary hydroxyl group, thereby suppressing undesired C─C bond cleavage while maintaining favorable reaction kinetics and improving selectivity toward GLA.

Building on these insights, we propose an integrated strategy that combines selective electrocatalytic oxidation of solketal with subsequent acidic hydrolysis to regenerate GLA (Scheme 1c). Using a non‐noble Cu_3_Mo_2_O_9_ catalyst with well‐defined and stable three‐dimensional Cu–O–Mo coordination frameworks, this approach enables highly efficient GLA electrosynthesis, delivering a record‐high selectivity of 90.1% and a GLA production rate of 675.45 µmol cm^−2^ h^−1^ at 1.40 V versus RHE. The system reaches ∼95% substrate conversion within 5 h and maintains stable performance over 20 consecutive cycles (totaling 100 h). Mechanistic investigations further reveal that highly alkaline conditions (6.0 M KOH) promote solketal deprotonation to reactive alkoxide species, weaken the C_α_─H bond to facilitate dehydrogenation, and buffer interfacial proton fluctuations to suppress local acidification, thereby stabilizing the ketal‐protected structure, preventing C─C bond cleavage, and ensuring high selectivity toward the GLA product. Finally, to demonstrate practical feasibility, we establish a proof‐of‐concept integrated upstream–downstream process, achieving solketal synthesis from glycerol and acetone in 90% yield (99% purity) and GLA isolation in 99% purity. Overall, this work highlights a substrate‐protection‐enabled strategy for selective C_3_ oxygenate production on non‐noble‐metal electrocatalysts, offering a promising route for sustainable glycerol valorization.

## 2 Results and Discussion

### 2.1 Synthesis and Characterization of the Cu_3_Mo_2_O_9_ Catalyst

**FIGURE 1:**
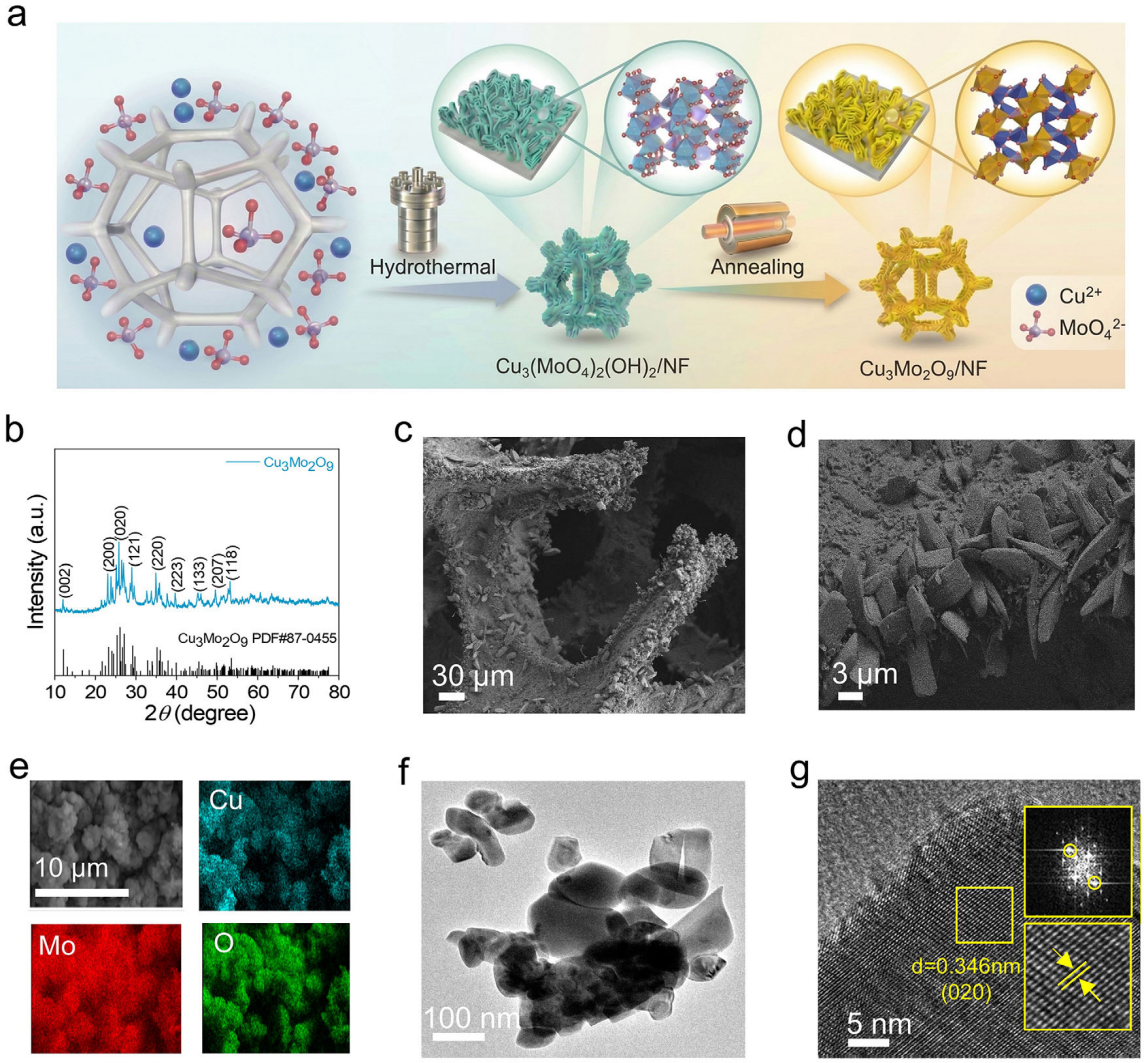
Synthesis and morphology of the Cu_3_Mo_2_O_9_ catalyst. (a) Schematic illustration of the preparation of Cu_3_Mo_2_O_9_/NF. (b) XRD pattern of Cu_3_Mo_2_O_9_. (c, d) SEM images of Cu_3_Mo_2_O_9_/NF. (e) EDS elemental mapping of Cu_3_Mo_2_O_9_/NF. (f) TEM image of Cu_3_Mo_2_O_9_. (g) HRTEM image of Cu_3_Mo_2_O_9_ (inset: FFT pattern and enlarged lattice fringes of the selected region).

The Cu_3_Mo_2_O_9_ catalyst electrode was synthesized through a two‐step procedure (Figure 1a). Nickel foam (NF) was employed as a porous and electrically conductive scaffold to support catalyst growth. In the first step, Cu^2+^ and molybdate (MoO_4_^2−^) precursors were hydrothermally deposited onto the NF substrate, affording a Cu_3_(MoO_4_)_2_(OH)_2_ intermediate (Figure S1). Subsequently, the as‐obtained precursor was annealed at 500°C for 2 h under an Ar atmosphere, promoting dehydration and crystallization to yield the final Cu_3_Mo_2_O_9_/NF electrode.

The crystal phase of the as‐synthesized catalyst was characterized by X‐ray diffraction (XRD) (Figure 1b). The Cu_3_Mo_2_O_9_ sample exhibits well‐defined diffraction peaks at 12.1°, 23.1°, 25.9°, 29.1°, 35.1°, 39.8°, 45.39°, 49.7°, and 53.2°, which can be indexed to the (002), (200), (020), (121), (220), (223), (133), (207), and (118) lattice planes of orthorhombic Cu_3_Mo_2_O_9_ (JCPDS 87‐0455), respectively. As illustrated in the Cu_3_Mo_2_O_9_ crystal structure (Figure 1a), Cu^2+^ ions adopt mixed octahedral (CuO_6_) and square‐pyramidal (CuO_5_) coordination geometries. In contrast, Mo^6+^ ions are tetrahedrally coordinated in MoO_4_ units, which corner‐share with neighboring CuO_6_ octahedra and CuO_5_ square pyramids [36], giving rise to an integrated three‐dimensional framework.

The morphology and microstructure of the Cu_3_Mo_2_O_9_/NF catalyst were further examined by scanning electron microscopy (SEM) and transmission electron microscopy (TEM). SEM images reveal that Cu_3_Mo_2_O_9_ nanostructures are uniformly anchored on the NF scaffold and feature an irregular, elliptical nanoplate‐like morphology (Figure 1c,d). Energy‐dispersive X‐ray spectroscopy (EDS) elemental mapping further confirms the homogeneous distribution of Cu, Mo, and O across the catalyst surface (Figure 1e). TEM observations corroborate the nanoplate‐like architecture of Cu_3_Mo_2_O_9_ (Figure 1f). The high‐resolution TEM (HRTEM) image displays clear lattice fringes with an interplanar spacing of 0.346 nm (Figure 1g), which is assigned to the (020) lattice plane of orthorhombic Cu_3_Mo_2_O_9_, consistent with the dominant diffraction peak observed in the XRD pattern (Figure 1b). This assignment is further supported by the corresponding fast Fourier transform (FFT) pattern (inset, Figure 1g).

**FIGURE 2:**
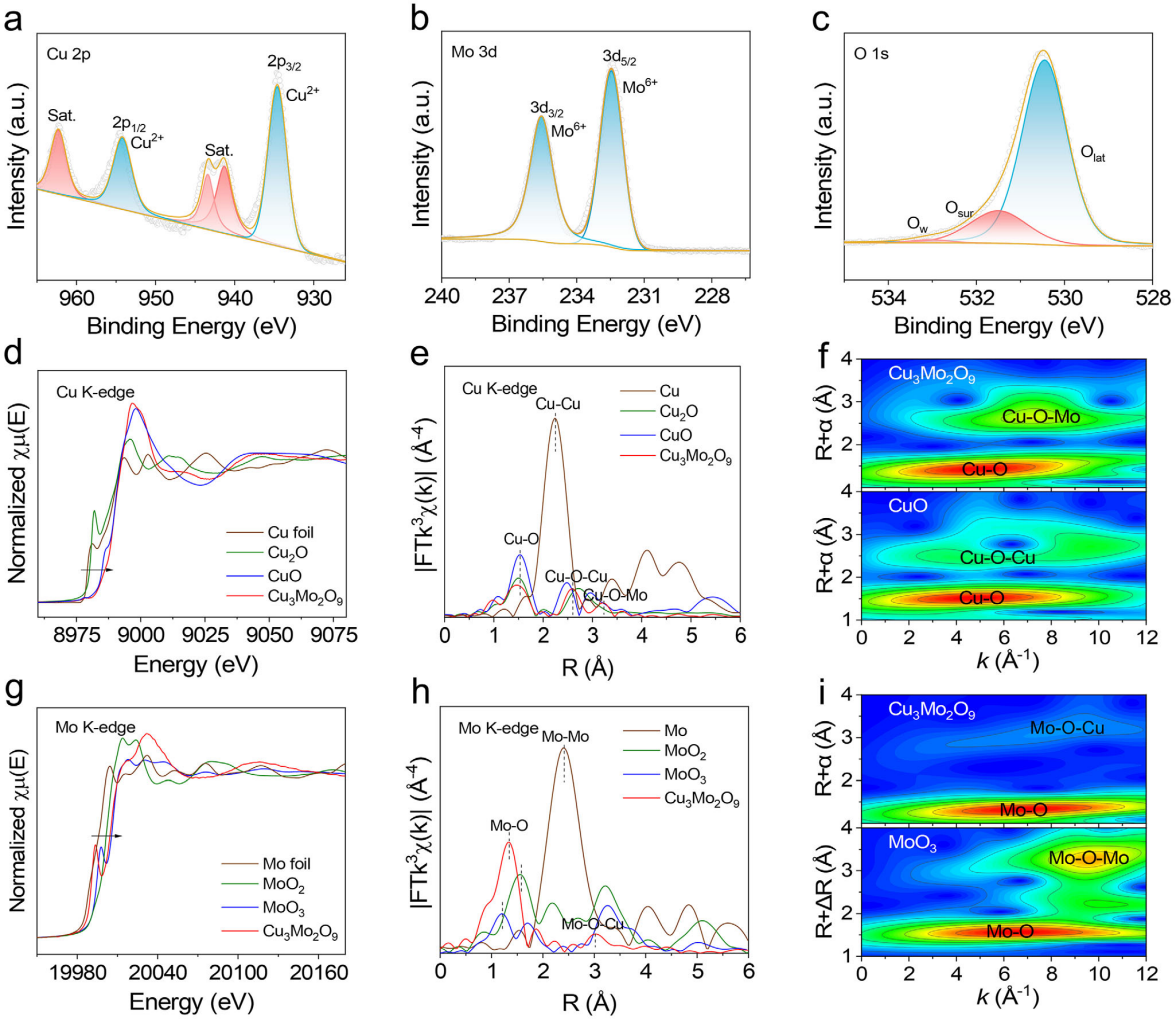
Structural characterization of the Cu_3_Mo_2_O_9_ catalyst. (a) Cu 2p, (b) Mo 3d, and (c) O 1s XPS spectra of Cu_3_Mo_2_O_9_. (d) Cu K‐edge XANES spectra of Cu_3_Mo_2_O_9_ and reference samples (Cu foil, Cu_2_O, and CuO). (e) Cu K‐edge FT‐EXAFS spectra in R‐space for Cu_3_Mo_2_O_9_ and reference samples (Cu foil, Cu_2_O, and CuO). (f) WT analysis of the Cu K‐edge EXAFS spectra of Cu_3_Mo_2_O_9_ and CuO. (g) Mo K‐edge XANES spectra of Cu_3_Mo_2_O_9_ and reference samples (Mo foil, MoO_2_, and MoO_3_). (h) Mo K‐edge EXAFS spectra in R‐space for Cu_3_Mo_2_O_9_ and reference samples (Mo foil, MoO_2_, and MoO_3_). (i) WT analysis of the Mo K‐edge EXAFS spectra of Cu_3_Mo_2_O_9_ and MoO_3_.

The elemental composition and surface chemical states of the Cu_3_Mo_2_O_9_/NF electrode were investigated by X‐ray photoelectron spectroscopy (XPS). The survey spectrum confirms the presence of Cu, Mo, and O elements (Figure S2), in good agreement with the EDS mapping results. The high‐resolution Cu 2p XPS spectrum can be deconvoluted into five peaks (Figure 2a): the peaks at 934.5 and 954.3 eV are assigned to Cu^2+^ 2p_3/2_ and 2p_1/2_ doublets, respectively, while the signals at 941.3, 943.4, and 962.3 eV correspond to the characteristic shake‐up satellite features of Cu^2+^ [37]. The Mo 3d XPS spectrum (Figure 2b) exhibits two distinct peaks at 232.4 and 235.6 eV, attributable to the Mo^6+^ 3d_5/2_ and 3d_3/2_ doublets [38]. The O 1s XPS spectrum (Figure 2c) is deconvoluted into three contributions at 530.6, 531.9, and 533.1 eV, corresponding to lattice oxygen (O_lat_), surface hydroxyl/adsorbed oxygen species (O_sur_), and adsorbed molecular water (O_w_), respectively [39].

To further elucidate the electronic structure and local coordination environments of Cu and Mo in Cu_3_Mo_2_O_9_, X‐ray absorption near‐edge structure (XANES) and extended X‐ray absorption fine structure (EXAFS) analyses were performed. The Cu K‐edge XANES spectrum of Cu_3_Mo_2_O_9_ (Figure 2d) displays a positive shift of the absorption edge relative to that of the CuO reference, suggesting a higher average oxidation state of Cu. The corresponding Fourier‐transformed EXAFS (FT‐EXAFS) spectrum at the Cu K‐edge in R space (Figure 2e) displays three distinct peaks at 1.47, 2.57, and 3.21 Å, which are attributed to the first‐shell Cu−O coordination, the Cu−O−Cu multiple‐scattering path, and the longer‐range Cu−O−Mo scattering contribution, respectively [40]. These assignments are further supported by wavelet transform (WT) analysis (Figure 2f and Figure S3). Compared with the WT contour plots of Cu foil and CuO, Cu_3_Mo_2_O_9_ exhibits two distinct intensity maxima at ∼5.05 and ∼7.20 Å^−1^, corresponding to Cu−O and Cu−O−Mo coordination contributions, respectively [41]. Quantitative fitting of the Cu K‐edge EXAFS data (Figure S4 and Table S1) gives an average coordination number (CN) of ∼5.92 for the Cu−O shell, confirming the coexistence of CuO_6_ and CuO_5_ polyhedra. Likewise, the Mo K‐edge XANES spectrum (Figure 2g) shows a slight negative edge shift compared to the MoO_3_ reference, indicative of a marginally increased electron density at the Mo centers. This behavior is attributed to electronic interactions within the Cu–O–Mo linkages [28]. Consistently, the Mo K‐edge FT‐EXAFS spectrum (Figure 2h) presents an intense peak at 1.44 Å arising from first‐shell Mo−O coordination, together with a second‐shell feature at ∼3.00 Å assigned to the reciprocal Mo−O−Cu scattering path [42, 43]. These structural assignments are further corroborated by WT‐EXAFS contour plots (Figure 2i and Figure S3). EXAFS fitting at the Mo K‐edge (Figure S4 and Table S1) reveals a Mo−O CN of ∼3.63, consistent with MoO_4_ tetrahedra. Collectively, the XANES and EXAFS results confirm a well‐defined Cu−O−Mo coordination network in Cu_3_Mo_2_O_9_, in good agreement with the structural information obtained from XRD and XPS analyses.

### 2.2 Electrocatalytic Performance of Cu_3_Mo_2_O_9_/NF for the Solketal Oxidation Reaction (SOR)

The electrocatalytic activity of Cu_3_Mo_2_O_9_/NF toward the SOR was evaluated in a standard three‐electrode H‐type divided cell. Given the critical influence of reactant and OH^−^ concentrations on the reaction kinetics of organic oxidation reactions, the electrolyte composition was first systematically optimized.

**FIGURE 3:**
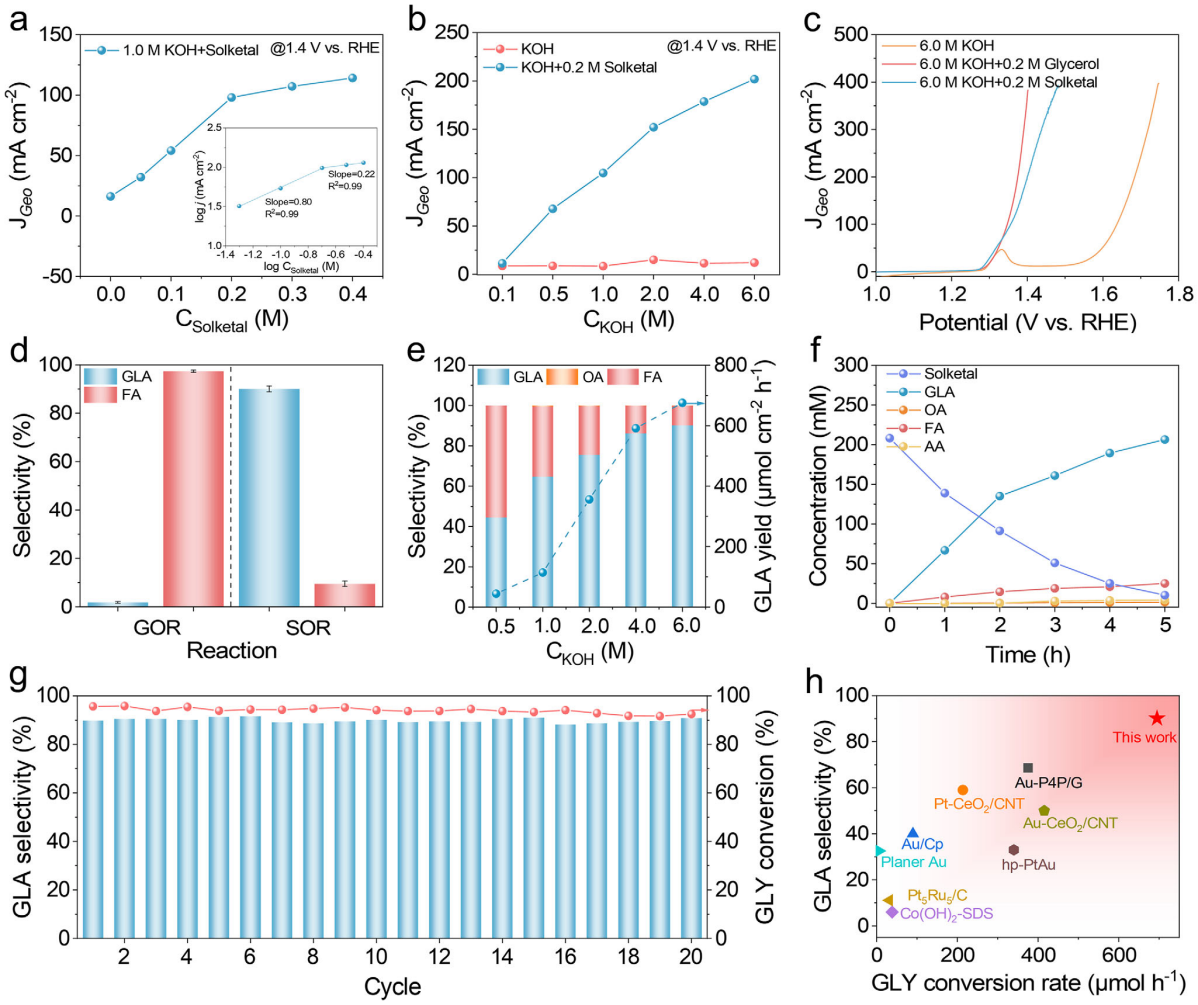
Solketal oxidation performance. (a) SOR current density as a function of solketal concentration in 1.0 M KOH; inset: log‐log plot used to determine the reaction order with respect to solketal. (b) SOR current density as a function of KOH concentration. (c) LSV profiles of Cu_3_Mo_2_O_9_/NF in 6.0 M KOH with and without 0.2 M glycerol or solketal. (d) Selectivities toward GLA and FA during GOR and SOR, with error bars derived from three independent parallel experiments. (e) Product selectivities and GLA yields at different KOH concentrations in the presence of 0.2 M solketal. (f) Product distribution during SOR. (g) GLA selectivity and glycerol (GLY) conversion over 20 consecutive electrolysis cycles (∼100 h). (h) Benchmark comparison of GLA selectivity and GLY conversion rate achieved in this work with values reported in previous studies.

The effect of solketal concentration was examined in 1.0 M KOH by varying the solketal concentration from 0.0 to 0.4 M (Figure 3a and Figure S5). At low solketal concentrations (≤ 0.2 M), the current density increased nearly proportionally with solketal concentration, giving a reaction order of 0.80 (inset, Figure 3a). However, further increasing the solketal concentration (> 0.2 M) led to a diminished enhancement in current density, with the apparent reaction order decreasing to 0.22. This suppression likely arises from excessive solketal adsorption at higher concentrations, which competitively limits the coverage of adsorbed OH* species required for oxidation [44]. Moreover, elevated solketal concentrations increase electrolyte viscosity and decrease ionic conductivity (Table S2), thereby impairing mass transport and charge transfer during electrocatalysis [45].

The influence of OH^−^ concentration was subsequently investigated by varying the KOH concentration from 0.1 to 6.0 M while maintaining a fixed solketal concentration of 0.2 M (Figure 3b and Figure S6). The current density increased monotonically with rising KOH concentration, indicating accelerated SOR kinetics under more alkaline conditions. This enhancement can be attributed not only to the increased availability of OH^−^ as the nucleophilic reactant, but also to the ability of concentrated alkaline electrolytes to sustain a high local pH at the electrode/electrolyte interface [46]. The higher OH^−^ concentration also promotes deprotonation of solketal to generate a more reactive alkoxide intermediate, according to the equilibrium: C_6_H_12_O_3_ + OH^−^ ⇌ C_6_H_11_O_3_^−^ + H_2_O [47, 48]. Overall, optimal SOR performance can be achieved by tuning solketal and OH^−^ concentrations to balance their interfacial co‐adsorption on the catalyst surface.

Representative linear sweep voltametry (LSV) profiles recorded in 6.0 M KOH electrolyte, with and without the addition of 0.2 M solketal or glycerol, are displayed in Figure 3c and Figure S7. The Cu_3_Mo_2_O_9_/NF electrode exhibits a markedly enhanced anodic response upon introduction of either solketal or glycerol, indicating that both SOR and GOR are kinetically favored over the oxygen evolution reaction (OER). Notably, a current density of 100 mA cm^−2^ for SOR is achieved at only 1.357 V versus RHE, which is 280 mV lower than that required to reach the same current density for OER. The SOR current density is slightly lower than that of GOR, likely because solketal contains only one oxidizable hydroxyl group, whereas glycerol possesses three active hydroxyl groups. Additionally, control experiments using CuO/NF, MoO_3_/NF, and bare NF (Figures S8 and S9) further demonstrate that Cu_3_Mo_2_O_9_/NF exhibits the highest anodic current and GLA yield, highlighting the advantage of the Cu–O–Mo coordination framework for SOR.

To clarify the critical role of vicinal hydroxyl protection in governing product selectivity, the product distributions from GOR and SOR were systematically compared after electrolysis at 1.40 V versus RHE for 2 h, based on three independent parallel experiments (Figure 3d and Figure S10). Liquid products were quantified by high‐performance liquid chromatography (HPLC) with a refractive index detector, using calibration curves obtained from standard solutions (Figure S11). For GOR, FA is the dominant product with an average selectivity of 97.3%, reflecting extensive C─C bond cleavage under strongly alkaline conditions on the Cu_3_Mo_2_O_9_ catalyst. In stark contrast, SOR predominantly yields GLA with a high selectivity of 90.1%, which is ∼50‐fold higher than that obtained in GOR (1.8%). These results demonstrate that ketal protection in solketal effectively suppresses C─C bond scission, thereby directing oxidation toward the unprotected primary hydroxyl group and enabling highly selective formation of the desired C_3_ product, GLA.

Followingly, the effect of KOH concentration on the product distribution during SOR was systematically investigated (Figure 3e and Figure S12). Electrolysis was conducted at a constant potential of 1.40 V versus RHE for 2 h. Increasing the KOH concentration from 0.5 to 6.0 M led to a continuous increase in both the yield and selectivity toward GLA, accompanied by a pronounced decrease in FA selectivity. In particular, in 6.0 M KOH, the GLA selectivity reached a maximum of 90.1% with a high GLA yield of 675.45 µmol cm^−2^ h^−1^ (Figure 3e). In contrast, at lower alkalinity (e.g., 0.5 M KOH), the SOR activity was substantially suppressed, affording a GLA selectivity of only 44.5% and a yield of 44.32 µmol cm^−2^ h^−1^, comparable to the FA selectivity (55.5%) and yield (55.32 µmol cm^−2^ h^−1^). These results emphasize the critical role of a highly alkaline environment in stabilizing the solketal C–C backbone and effectively steering the oxidation pathway toward selective GLA formation, which will be discussed in detail in the following mechanistic elucidation section.

Furthermore, the potential‐dependent product distribution was investigated (Figure S13). The solketal conversion, quantified from the glycerol peak in HPLC after acid hydrolysis (Figure S14), increased steadily as the applied potential was raised from 1.35 to 1.60 V versus RHE, with GLA remaining the dominant product (selectivity > 77%). However, at potentials above 1.40 V versus RHE, the GLA selectivity began to decline, while the formation of C_1_ and C_2_ products (e.g., FA, OA, and AA) became more pronounced (Figure S13b), indicative of enhanced overoxidation and C─C bond cleavage at higher potentials. In particular, AA was detected at 1.60 V versus RHE, which is likely associated with the oxidation of acetone generated by solketal hydrolysis under strongly oxidative conditions (Figures S15 and S16).

To survey the catalytic performance of the Cu_3_Mo_2_O_9_/NF electrode during continuous SOR, reactant consumption and product generation were monitored during electrolysis at 1.40 V versus RHE (Figure 3f and Figure S17). As the solketal concentration progressively decreased over time, the concentration of the primary product, GLA, increased continuously, whereas by‐products such as OA, AA, and FA remained at low concentration levels throughout the reaction. After 5 h of electrolysis, the solketal conversion exceeded 95%, and the GLA yield reached 1.96 mmol (Figure 3f). The long‐term durability of Cu_3_Mo_2_O_9_/NF was further assessed over 20 consecutive electrolysis cycles (5 h per cycle, 100 h total) at 1.40 V versus RHE (Figure 3g and Figure S18). Throughout the cycling tests, the catalyst sustained stable GLA production with an average yield of ∼1.93 mmol per cycle, along with an average GLA selectivity of 89.84% and solketal conversion of 93.97%, demonstrating excellent operational stability (Table S3). Notably, post‐electrolysis characterizations of the catalyst, including XRD, SEM, TEM, and XPS, reveal negligible changes in the crystal structure, surface morphology, and chemical states of Cu_3_Mo_2_O_9_ (Figures S19–S21). Furthermore, inductively coupled plasma mass spectrometry (ICP‐MS) analysis confirms negligible metal leaching into the electrolyte during prolonged continuous operation, which is significantly lower than that observed for the GOR process (Table S4). Collectively, these results corroborate the exceptional structural robustness and compositional stability of the Cu_3_Mo_2_O_9_ catalyst under SOR conditions.

Finally, the performance of Cu_3_Mo_2_O_9_/NF was benchmarked against recently reported electrocatalysts for electrochemical GLA production (Figure 3h and Table S5). As a low‐cost, non‐noble metal catalyst, Cu_3_Mo_2_O_9_ achieves a record‐high GLA selectivity of 90.1% and a glycerol (solketal) conversion rate of 695.21 µmol h^−1^, outperforming state‐of‐the‐art precious‐metal‐based systems to the best of our knowledge.

### 2.3 Mechanistic Insights Into SOR Under Varying KOH Concentrations

**FIGURE 4:**
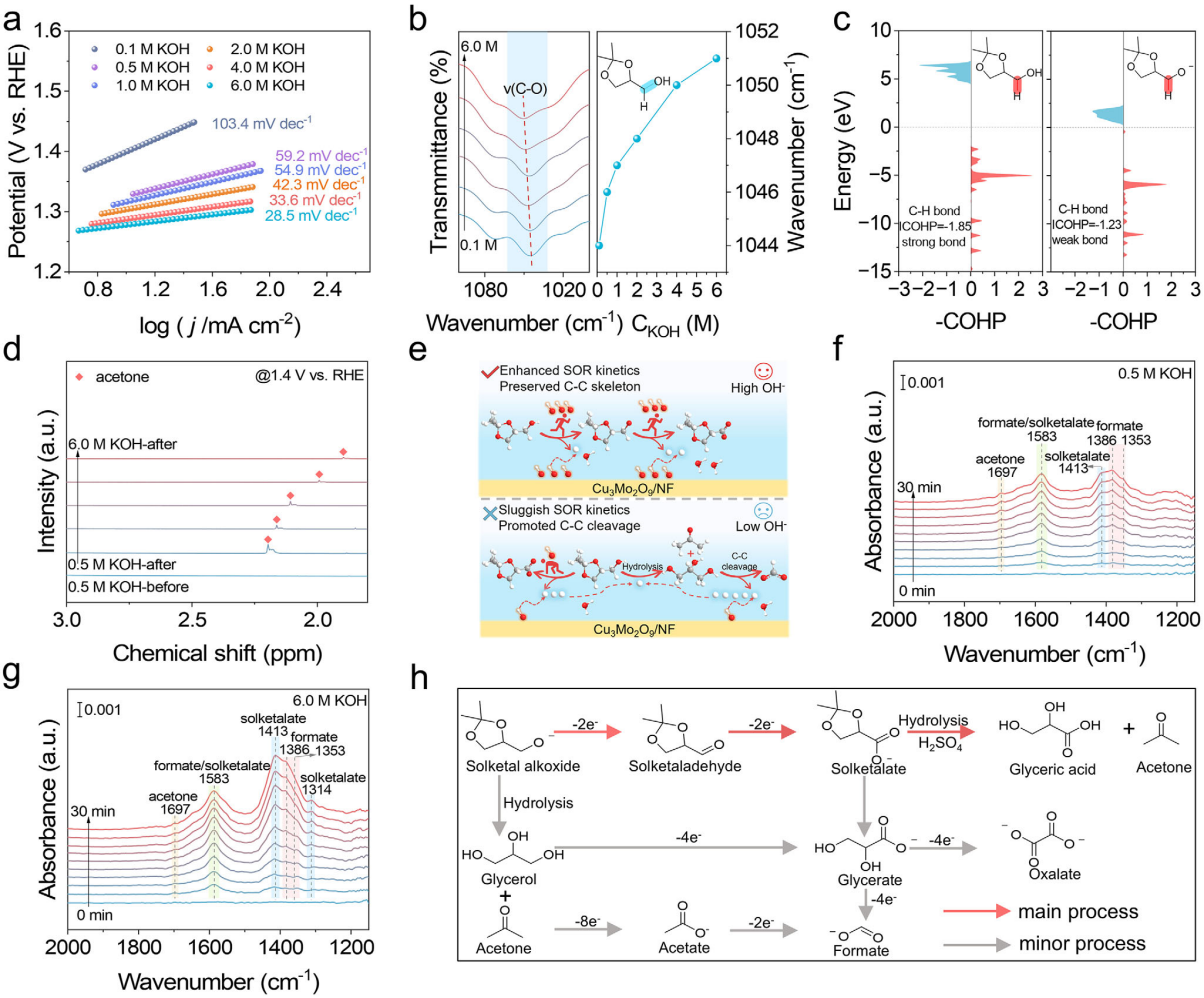
Mechanistic elucidation. (a) Tafel plots of Cu_3_Mo_2_O_9_/NF in different KOH concentrations containing 0.2 M solketal. (b) FTIR spectra of solketal in varying KOH concentrations (left) and the corresponding *v*_C−O_ shift (right). (c) COHP analysis of the C_α_─H bond in solketal (left) and its alkoxide form (right). (d) ^1^H NMR spectra of electrolytes collected after SOR at 1.40 V versus RHE for 2 h in different KOH concentrations with 0.2 M solketal, compared with the spectrum of the initial 0.5 M KOH electrolyte before electrolysis. (e) Schematic illustration of the proposed mechanism in concentrated and dilute KOH electrolytes. (f, g) Time‐resolved in situ ATR‐FTIR spectra recorded during SOR at 1.40 V versus RHE in 0.5 M KOH (f) and 6.0 M KOH (g). (h) Proposed reaction pathways for SOR in alkaline electrolyte.

To gain a deeper understanding of how OH^−^ concentration regulates both the electrocatalytic activity of SOR and the selectivity toward GLA, we further investigated the reaction kinetics and mechanistic pathway. The kinetics of SOR on Cu_3_Mo_2_O_9_/NF at different KOH concentrations were first evaluated using Tafel analysis (Figure 4a). The extracted Tafel slopes decrease from 103.4 to 28.5 mV dec^−1^ as the KOH concentration increases from 0.1 to 6.0 M (0.2 M solketal), with values of 103.4, 59.2, 54.9, 42.3, 33.6, and 28.5 mV dec^−1^ for 0.1, 0.5, 1.0, 2.0, 4.0, and 6.0 M KOH, respectively. The substantially smaller Tafel slopes in concentrated alkaline electrolytes indicate markedly accelerated SOR kinetics under more alkaline conditions. This enhancement can be attributed to an increased interfacial OH^−^ concentration, which facilitates solketal deprotonation and the formation of a more reactive alkoxide intermediate (C_6_H_11_O_3_^−^) [47].

To verify the formation of an alkoxide intermediate, Fourier transform infrared (FTIR) spectroscopy was conducted on Cu_3_Mo_2_O_9_/NF in electrolytes with different KOH concentrations in the presence of 0.2 M solketal (Figure 4b; left). With increasing KOH concentration, the characteristic C−O stretching vibration of the CH_2_OH group in solketal (*v*_C−O_) [9] gradually shifts from 1044 cm^−1^ in 0.1 M KOH to 1051 cm^−1^ in 6.0 M KOH (Figure 4b; right). This blue shift to a higher wavenumber is attributed to increased electron density on the oxygen atom of the C─O bond [46]. This observation implies that elevated OH^−^ concentration promotes deprotonation of the solketal hydroxyl group, thereby facilitating the formation of reactive alkoxide species and enhancing subsequent proton abstraction from the C_α_─H bond of the CH_2_OH moiety during SOR [46, 48].

To rationalize the enhanced reactivity of the alkoxide species, crystal orbital Hamilton population (COHP) analysis was performed for solketal and its alkoxide form [49]. The integrated COHP (ICOHP) value for the C_α_─H bond in the CH_2_OH moiety of solketal is −1.85, whereas that of the corresponding alkoxide species (C_6_H_11_O_3_^−^) increases to −1.23 (Figure 4c). The less negative ICOHP value indicates a weakened C_α_─H bond upon alkoxide formation, which facilitates C_α_−H cleavage and lowers the kinetic barrier for the dehydrogenation step toward solketaladehyde (i.e., GLAD with acetone protection). Further insight into the C_α_─H bonding characteristics of solketal and its alkoxide was obtained from electron localization function (ELF) analysis (Figure S22) [50]. Solketal exhibits pronounced electron localization between the C and H atoms, consistent with a strong covalent interaction, whereas the alkoxide (C_6_H_11_O_3_^−^) shows more delocalized electron density in the C_α_─H region, supporting a weakened C_α_─H bond. These ELF results corroborate the COHP analysis, confirming that alkoxide formation weakens the C_α_─H bond, promotes dehydrogenation, and ultimately accelerates SOR kinetics.

Furthermore, given OH^−^ is continuously consumed during SOR, local acidification at the electrode/electrolyte interface may occur, which could promote solketal hydrolysis and thus impact product selectivity [46, 51]. At high KOH concentrations, the abundant OH^−^ effectively buffers against local pH fluctuations, preserving the acetone‐protected glycerol backbone and favoring selective GLA formation. In contrast, under dilute alkaline conditions, depletion of OH^−^ may lead to interfacial acidification, facilitating solketal deprotection to glycerol and acetone. The resulting glycerol and acetone can then undergo their respective oxidation pathways to form FA and AA (Figures S10 and S15). Although direct measurement of local pH was not feasible, this effect was indirectly assessed by tracking the formation of hydrolysis‐derived acetone during the electrolysis process. Proton nuclear magnetic resonance (^1^H NMR) spectroscopy was employed to monitor acetone in electrolytes at different KOH concentrations (Figure 4d and Figure S23). A characteristic acetone resonance at ∼2.19 ppm, assigned to its methyl protons, emerges after 2 h of electrolysis in 0.5 M KOH, whereas no acetone signal is observed in the initial solution (0 h), confirming that solketal is stable under alkaline conditions in the absence of electrolysis but undergoes partial hydrolysis during SOR. Notably, increasing the KOH concentration from 0.5 to 6.0 M leads to a progressive decrease in acetone signal intensity, accompanied by an upfield shift (from 2.19 to 1.85 ppm) after 2 h of electrolysis. The upfield shift is attributed to electrolyte‐induced solvent effects that enhance magnetic shielding of the methyl protons [52], as supported by control experiments (Figure S24). More importantly, the diminished signal intensity indicates that higher OH^−^ concentrations effectively suppress solketal hydrolysis. These results confirm that acetone formation arises from acid‐activated ketal deprotection under dilute KOH conditions during electrolysis. Conversely, in concentrated KOH (6.0 M), hydrolysis of solketal is largely inhibited, thus preserving the ketal structure and ensuring high selectivity toward GLA.

As discussed above, the enhanced activity and GLA selectivity for SOR at elevated KOH concentrations can be ascribed to two key factors, as schematically illustrated in Figure 4e. First, the highly alkaline environment promotes deprotonation of the solketal hydroxyl group, generating reactive alkoxide species. Alkoxide formation weakens the C_α_─H bond and facilitates the subsequent dehydrogenation step, thereby markedly accelerating SOR kinetics. Second, the high OH^−^ concentration effectively buffers protons and mitigates local acidification at the electrode/electrolyte interface. As a result, the ketal structure is preserved, allowing selective oxidation of the exposed primary hydroxyl group to a carboxyl group and ultimately enabling highly selective formation of the value‐added C_3_ product GLA using a non‐noble‐metal catalyst system.

To further probe the dynamic reaction pathways of SOR catalyzed by Cu_3_Mo_2_O_9_/NF, in situ attenuated total reflectance Fourier transform infrared (ATR‐FTIR) spectroscopy was performed (setup shown in Figure S25). Electrolysis was conducted at 1.40 V versus RHE in either 0.5 or 6.0 M KOH containing 0.2 M solketal, and time‐resolved spectra were collected over a period of 0−30 min. Reference infrared spectra of solketal, glycerol, acetone, GLA, FA, and OA in KOH solution are provided in Figure S26. As shown in Figure 4f,g, the band at 1583 cm^−1^ is assigned to the asymmetric stretching vibration of carboxylate groups (*v*_O−C = O_) [44], indicating the formation of carboxylate‐containing intermediates/products such as solketalate (acetone‐protected GLA) and FA. The peaks at 1314 and 1413 cm^−1^ are attributed to C−O stretching and symmetric O−C−O stretching vibrations of solketalate [51, 53], respectively, providing direct evidence for the selective oxidation of the primary hydroxyl group of solketal to a carboxylate. In addition, the bands at 1353 and 1386 cm^−1^ correspond to the symmetric O−C−O stretching vibrations of FA [54]. The appearance of FA‐related features is consistent with ketal deprotection of solketal to glycerol, followed by subsequent glycerol oxidation through C–C cleavage pathways. Furthermore, the emergence of a band at 1697 cm^−1^, characteristic of the C═O stretching vibration of acetone [55], confirms hydrolytic deprotection of the ketal moiety during electrolysis, particularly under dilute alkaline conditions (0.5 M KOH). Notably, spectra acquired in 6.0 M KOH exhibit stronger solketal‐associated signals and markedly weaker acetone signals (Figure 4g and Figure S27), suggesting that highly alkaline conditions favor selective oxidation of the exposed primary hydroxyl group while largely preserving ketal protection. In contrast, spectra recorded in 0.5 M KOH show diminished solketalate signals together with pronounced acetone features (Figure 4f), indicative of suppressed SOR activity and enhanced ketal deprotection. This behavior is consistent with local acidification at the electrode/electrolyte interface during electrolysis under insufficiently buffered alkaline conditions.

Based on the results and discussions presented above, a plausible reaction pathway for SOR catalyzed by Cu_3_Mo_2_O_9_/NF is proposed and summarized in Figure 4h. In the dominant pathway (red arrows), solketal is first deprotonated in the highly alkaline electrolyte to generate reactive solketal alkoxide species. The alkoxide then adsorbs on the catalyst surface and undergoes a two‐electron dehydrogenation to form a solketaldehyde intermediate [20]. The aldehyde is subsequently oxidized to solketalate, which, upon acidification, yields GLA as the primary product [56]. In a competing pathway (gray arrows), partial hydrolysis of solketal induces ketal deprotection, producing glycerol and acetone. The resulting unprotected glycerol is readily oxidized under alkaline conditions, undergoing C─C bond cleavage to yield FA (C_1_) and OA (C_2_) [57, 58]. Acetone, on the other hand, requires prolonged electrolysis and higher anodic potentials (≥ 1.60 V vs. RHE) to be oxidized to AA and FA (Figure S16) [55]. Notably, under highly alkaline conditions (6.0 M KOH), direct oxidation of solketal to solketalate is overwhelmingly favored, delivering > 90% selectivity toward GLA. This selective pathway effectively suppresses ketal‐deprotection routes, underscoring the crucial role of electrolyte alkalinity in steering reaction pathways and enabling efficient solketal valorization to value‐added GLA.

### 2.4 Process Integration: Solketal Synthesis, GLA Isolation, and Economic Assessment

**FIGURE 5:**
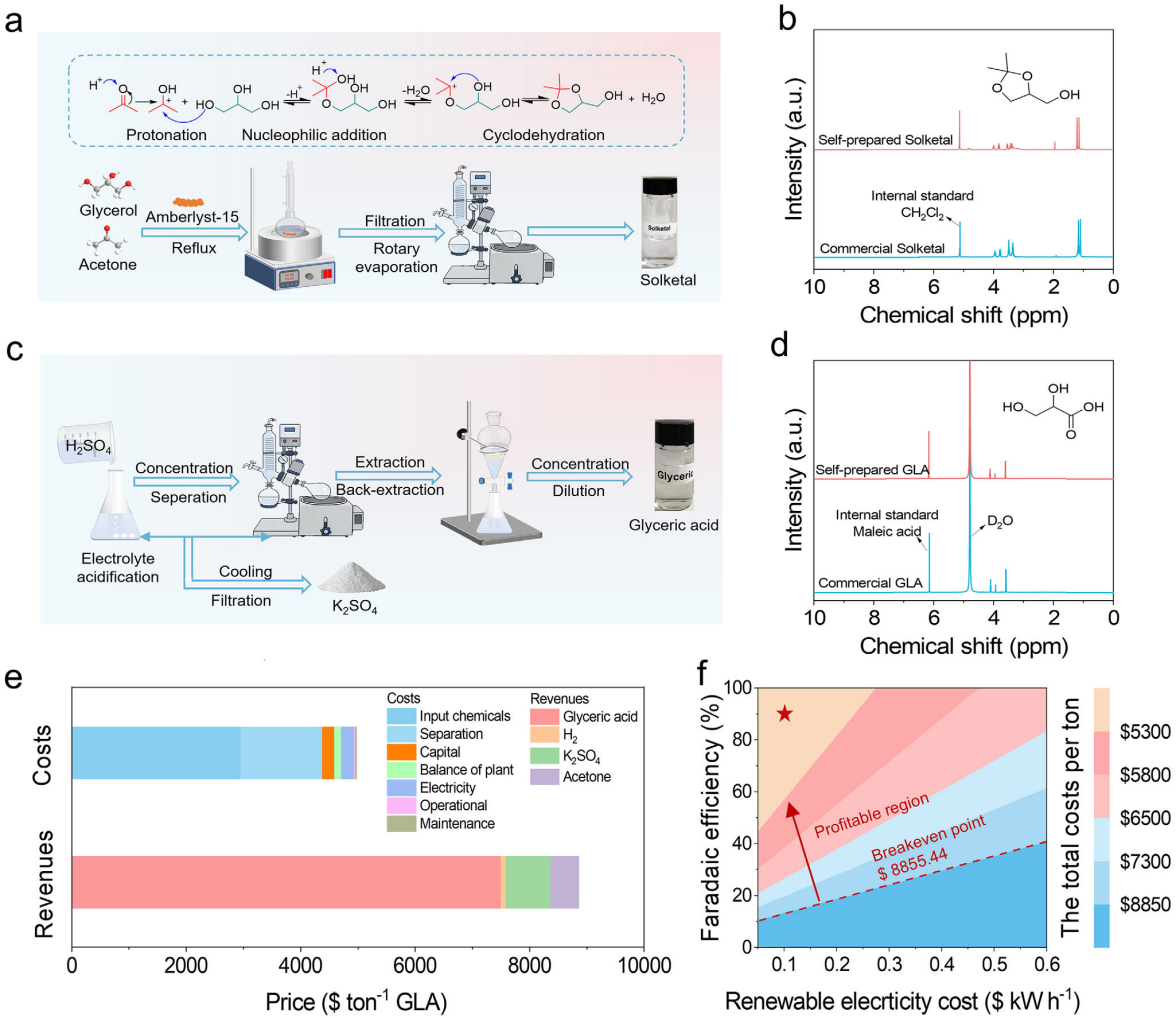
Process integration and techno‐economic analysis. (a) Schematic illustration of the acid‐catalyzed synthesis of solketal. (b) ^1^H NMR spectra of the as‐synthesized solketal and a commercial solketal standard. (c) Schematic illustration of the downstream separation and purification of the GLA product. (d) ^1^H NMR spectra of the as‐synthesized GLA and a commercial GLA standard. (e) Cost and revenue analysis of GLA electrosynthesis. (f) Techno‐economic analysis of GLA electrosynthesis.

To reduce feedstock costs and improve overall process feasibility, an efficient acid‐catalyzed route for solketal synthesis was developed. As illustrated in Figure 5a, the reaction proceeds *via* a classical ketalization mechanism [59]. Briefly, protonation of the acetone carbonyl group first generates a reactive oxonium ion, increasing the electrophilicity of the carbonyl carbon. Nucleophilic attack by the primary hydroxyl group of glycerol then affords a hemiketal intermediate, which undergoes protonation and dehydration to form a tertiary carbocation. Subsequent intramolecular nucleophilic attack by the adjacent secondary hydroxyl group induces cyclization, followed by dehydration to yield the five‐membered cyclic ketal, 1,2‐isopropylidene glycerol (solketal) [60]. Experimentally, glycerol (9.209  g) and acetone (17.424  g) were reacted under reflux in a 60°C oil bath for 6 h using Amberlyst‐15 (4.600  g) as a solid acid catalyst. After completion of the reaction, the Amberlyst‐15 catalyst was removed by filtration and recovered for reuse. Excess acetone was eliminated under reduced pressure using rotary evaporation. The chemical structure of the as‐synthesized solketal (11.898 g, 90% yield) was confirmed by ^1^H NMR spectroscopy (99% impurity, Figure 5b) and FTIR analysis (Figure S28), both of which are fully consistent with those of a commercial solketal standard, verifying the successful synthesis of high‐purity solketal suitable for subsequent electrooxidation studies.

To further validate the commercial viability of electrochemical SOR for the production of value‐added GLA, the downstream separation and purification process was systematically investigated (Figure 5c). The mixed electrolyte collected after consecutive electrolysis (Figure 3g) was first acidified with 3.0 M H_2_SO_4_, hydrolyzing solketalate into GLA and acetone while simultaneously precipitating K_2_SO_4_, which was recovered by filtration. The filtrate was then concentrated under reduced pressure to remove most of the water and residual acetone. Subsequent cooling induced further crystallization of K_2_SO_4_, and the recovered solid phase was confirmed by XRD (Figure S29). After complete removal of the precipitated K_2_SO_4_, the remaining filtrate—composed primarily of GLA with trace amounts of FA and glycerol—was adjusted to pH ∼11 to enable boronic‐acid‐mediated reactive extraction [61]. The aqueous phase was contacted with an organic phase consisting of n‐hexane/n‐octanol (85:15, v/v) containing 2‐naphthaleneboronic acid (2‐NB) and tri‐octyl methyl ammonium chloride (TOMAC). Here, 2‐NB selectively formed a boronate ester with the vicinal diol moiety of GLA, which subsequently associated with TOMAC to generate a hydrophobic ion pair that was extracted into the organic phase, while glycerol and FA remained in the aqueous phase. The GLA‐loaded organic phase was back‐extracted with dilute HCl, followed by gradient reduced‐pressure rotary evaporation to remove most of the solvents. To further eliminate residual moisture, the sample was subjected to lyophilization at −25°C under high vacuum (10 Pa) for 48 h. Despite this rigorous drying process, the final product remained a pale‐yellow, viscous liquid, consistent with its inherently strong hygroscopic nature, and exhibited an organic purity of 99% on a dry organic basis (Figure S30). Given that neat GLA is highly hygroscopic, poorly crystalline, and exhibits limited storage stability, the product was diluted to a ∼20 wt% aqueous solution for practical handling and downstream applications. The identity and purity of the final product were confirmed by ^1^H NMR spectroscopy (Figure 5d), HPLC analysis (Figure S31a), and FTIR spectroscopy (Figure S31b), all of which are in excellent agreement with a commercial 20% GLA solution standard.

To evaluate the economic viability and industrialization potential of sustainable GLA electrosynthesis using glycerol and acetone as feedstocks, a techno‐economic analysis (TEA) was performed based on a modified model proposed by Sargent (Figure S32, details provided in the Supporting Information) [62, 63, 64]. The analysis accounts for total operating and capital‐related costs, including feedstocks, capital expenditure, electricity consumption, and maintenance, as well as revenues associated with product generation (Figure 5e). Under the assumed operating conditions (current density of 100 mA cm^−2^, cell voltage of 1.75 V, 90% FE, and electricity cost of $0.1 kWh^−1^, Figure S33 and Table S6), the Cu_3_Mo_2_O_9_/NF‐catalyzed electrochemical SOR to GLA, coupled with H_2_ evolution at the cathode, delivers an estimated revenue of ∼$8855.44 per ton of GLA. This value substantially exceeds the corresponding production cost of $4919.27 per ton (Figure 5e), resulting in a projected net profit of $3936.17 per ton of GLA (indicated by the star in Figure 5f). Sensitivity analysis further reveals a wide profitable operating window across varying electricity prices and FEs (Figure 5f), with a breakeven point of $8855.44 per ton of GLA. Collectively, these results highlight the strong economic competitiveness and industrial promise of the integrated electrocatalytic SOR process as a sustainable route for GLA production. Nevertheless, this TEA serves as a prospective baseline assessment. Practical implementation will rely on “scaling out” via modular electrolyzer stacks, such as zero‐gap membrane electrode assemblies. Specifically, mitigating Ohmic losses and mass transport limitations, as well as optimizing thermal management in large‐area electrolyzers, will be critical to preserving the reported performance at scale.

## 3 Conclusion

In summary, we establish a substrate‐protection‐enabled electrocatalytic strategy that overcomes the long‐standing selectivity limitation of non‐noble catalysts in glycerol oxidation, where extensive C─C bond cleavage typically leads to low‐value C_1_ products. By replacing glycerol with its ketal‐protected derivative, solketal, the rigid five‐membered ketal ring sterically shields the vicinal C_2_ and C_3_ hydroxyl groups while selectively exposing the primary C_1_ hydroxyl group, thereby enforcing site‐selective oxidation and suppressing undesired C–C scission at the molecular level. Using a non‐noble metal Cu_3_Mo_2_O_9_ catalyst, the SOR delivers a record‐high GLA selectivity of 90.1% with a production rate of 675.45 µmol cm^−2^ h^−1^, while maintaining excellent operating stability over 100 h. Mechanistic studies reveal that highly alkaline electrolytes (6.0 M KOH) simultaneously enhance activity and preserve selectivity by promoting solketal deprotonation to reactive alkoxide species, weakening the C_α_─H bond to facilitate the dehydrogenation step, and buffering interfacial proton fluctuations to mitigate local acidification and electrolysis‐induced ketal hydrolysis. Beyond catalytic performance, we demonstrate a proof‐of‐concept integrated upstream–downstream process, achieving solketal synthesis from glycerol and acetone in 90% yield and isolating GLA with 99% purity, while TEA indicates a broad profitable operating window under practical electricity prices and Faradaic efficiencies. Overall, this study introduces substrate‐level molecular protection as a generalizable paradigm for pathway steering and C─C bond preservation in a non‐noble catalyst system. This concept is expected to be extendable to other multifunctional biomass‐derived polyol molecules, providing a versatile and sustainable route for the selective electrosynthesis of value‐added oxygenates using earth‐abundant catalysts and renewable electricity.

## Author Contributions

**Jiamin Wang**: writing – original draft, formal analysis, methodology, visualization. **Baokun Zhang**: writing – original draft, formal analysis, methodology, visualization. **Kanglei Pang**: writing – original draft, formal analysis, methodology, visualization. **Egon Campos dos Santos**: data curation, software, writing – review and editing, formal analysis, methodology. **Hong Liu**: writing – review and editing, conceptualization, project administration, supervision, formal analysis, data curation, funding acquisition, validation. **Mats Johnsson**: writing – review and editing, supervision, formal analysis, validation, methodology. **Xiaowen Yu**: conceptualization, writing – review and editing, project administration, supervision, formal analysis, data curation, funding acquisition, validation, methodology.

## Conflicts of Interest

The authors declare no conflicts of interest.

## Supporting information




**Supporting File**: anie73174‐sup‐0001‐SuppMat.pdf.

## Data Availability

The data that support the findings of this study are available from the corresponding author upon reasonable request.
