# Supplementary material for: Ketal Protection of Glycerol for Selective Electrosynthesis of Glyceric Acid in Highly Alkaline Media
Source: Angew Chem Int Ed Engl. 2026 Jun 18;65(36):e5555743. doi: 10.1002/anie.5555743 (PMC13528518; doi:10.1002/anie.5555743)
Supplement: Supplementary file 1 [file ANIE-65-e5555743-s001.pdf]

Supporting Information

Ketal Protection of Glycerol for Selective Electrosynthesis of Glyceric Acid in Highly Alkaline Media

Jiamin Wang,[a] Baokun Zhang,[a] Kanglei Pang,[b] Egon Campos dos Santos,[c] Hong Liu,*[a] Mats Johnsson,*[b] and Xiaowen Yu*[a]

[a] J. Wang, B. Zhang, Prof. H. Liu, Prof. X. Yu
State Key Laboratory of Crystal Materials, Shandong University, Jinan 250100, P. R. China
E-mail: hongliu@sdu.edu.cn (H. Liu), xiaowen.yu@sdu.edu.cn (X. Yu)

[b] Dr. K. Pang, Prof. M. Johnsson
Department of Chemistry, Stockholm University, SE-10691, Stockholm, Sweden
E-mail: mats.johnsson@su.se (M. Johnsson)

[c] Dr. E.C. dos Santos
Departamento de Ciências Naturais, Universidade Federal de São João del-Rei, 36301-160 São João del-Rei, MG, Brazil

Experimental Section

Chemicals and Materials

Sodium molybdate dihydrate ($Na_2MoO_4·2H_2O$, Macklin, 99%), copper(II) sulfate pentahydrate ($CuSO_4·5H_2O$, Macklin, 99%), DL-1,2-isopropylideneglycerol (solketal, Aladdin, ≥98%), potassium hydroxide (KOH, Macklin, 95%), sulfuric acid (H_2SO_4, Kermel, AR), glycerol (Macklin, ≥99.5%), oxalic acid (Sigma-Aldrich, ≥98%), DL-glyceric acid (TCI, 20% in water, 2 M), formic acid (Sigma-Aldrich, reagent grade, ≥95%), glacial acetic acid (Macklin, ≥99.8%), acetone (Yantai Far East Fine Chemical Co., Ltd., AR), Amberlyst 15 ion-exchange resin (Bidepharm, H^+ ≥ 4.7 mmol/g) were all used as obtained without further purification. Nickel foam (NF) was purchased from SCI Materials Hub. The ultrapure water from the UPHW-III-90T system was used to prepare all aqueous solutions.

Preparation of $Cu_3Mo_2O_9$/NF

Prior to synthesis, nickel foam (NF, 2 × 6 cm^2) substrates were ultrasonically cleaned in 3 M HCl for 15 min to remove surface oxides, followed by thorough rinsing with deionized (DI) water (four times, 15 min each). Subsequently, 6 mmol of $Na_2MoO_4·2H_2O$ and 6 mmol of $CuSO_4·5H_2O$ were separately dissolved in 60 mL of DI water. The $Na_2MoO_4·2H_2O$ solution was then slowly added dropwise to the $CuSO_4·5H_2O$ solution under vigorous magnetic stirring for 1 h to obtain a homogeneous precursor mixture. The resulting solution was transferred into a 100 mL Teflon-lined stainless-steel autoclave, into which a piece of the pre-treated NF substrate was immersed. The hydrothermal reaction was conducted at 160 °C for 12 h. After naturally cooling to room temperature, the NF substrate covered with light-green precipitates was removed, thoroughly washed with DI water to eliminate residual reactants, and then freeze-dried for 12 h. The obtained $Cu_3(MoO_4)_2(OH)_2$/NF precursor was subsequently calcined at 500 °C for 2 h under an Ar atmosphere with a heating rate of 2 °C min^{-1} to yield $Cu_3Mo_2O_9$/NF. After annealing, the 2 × 6 cm^2 $Cu_3Mo_2O_9$/NF electrode was cut into small electrodes with a geometric surface area of 1 × 1 cm^2 for electrochemical measurements.

Preparation of CuO/NF and MoO_3/NF

The CuO/NF control sample was synthesized following a procedure analogous to that of $Cu_3Mo_2O_9$/NF, except that $Na_2MoO_4·2H_2O$ was omitted. Briefly, aqueous ammonia was added dropwise to an aqueous $CuSO_4·5H_2O$ solution under continuous stirring until a clear solution was obtained, signifying the formation of a soluble copper-ammine complex. The resulting

solution was then subjected to the same hydrothermal treatment and subsequent calcination in air as described for $Cu_3Mo_2O_9$/NF, yielding the CuO/NF electrode.

For the MoO_3/NF control sample, the synthesis was carried out in the absence of $CuSO_4·5H_2O$. To facilitate hydrolysis and polymerization of molybdate species, dilute hydrochloric acid (1.0 M) was added dropwise under vigorous stirring to adjust the solution pH to approximately 1. The mixture was then subjected to identical hydrothermal treatment and calcination in air, affording the MoO_3/NF electrode.

Characterization

The morphology and microstructure of the samples were examined using scanning electron microscopy (SEM, Geimini 500, Carl Zeiss, Germany) equipped with an energy-dispersive X-ray spectroscopy (EDS) detector. High-resolution scanning transmission electron microscopy (HR-STEM) images were taken on a JEM-F200 microscope (JEOL, Tokyo, Japan) operated at an accelerating voltage of 200 kV. Powder X-ray diffraction (XRD) patterns were recorded on a Bruker D8 ADVANCE diffractometer with Cu Kα radiation (λ = 1.5406 Å) at 40 kV and 40 mA. X-ray photoelectron spectroscopy (XPS) measurements were performed on a Thermo Scientific ESCALAB 250Xi XPS instrument using Al kα radiation, with a spot size of 500 μm. Survey spectra were recorded with a pass energy of 150 eV, while high-resolution spectra were collected with a pass energy of 30 eV. X-ray absorption near-edge structure (XANES) and extended X-ray absorption fine structure (EXAFS) spectra were recorded in transmission mode at the Balder beamline of the MAX IV Laboratory, Sweden. All samples were calibrated using a Mn foil. Data analysis was performed using the Athena software. The dissolved metal content in the electrolyte after long-term testing was quantified by inductively coupled plasma mass spectrometry (ICP-MS, NexION 350X).

Electrochemical Measurements

Electrochemical measurements were performed at room temperature using a CHI-660E electrochemical workstation (Shanghai Chenhua Instrument Company) in a three-electrode configuration. A standard H-type two-compartment glass cell was employed, in which the cathodic and anodic compartments were separated by a Nafion 211 membrane. A graphite rod and a Hg/HgO (1.0 M KOH) electrode were used as the counter electrode and reference electrodes, respectively, while $Cu_3Mo_2O_9$/NF (1 cm × 1 cm) served as the working electrode. For the solketal oxidation reaction (SOR), the electrolyte consisted of KOH solutions at various concentrations (0.1, 0.5, 1.0, 2.0, 4.0, and 6.0 M) with or without 0.2 M solketal. For the glycerol oxidation reaction (GOR), the electrolyte was 6.0 M KOH with or without 0.2 M glycerol. Cyclic

voltammetry (CV) and linear sweep voltammetry (LSV) measurements were conducted at a scan rate of 10 mV s^{-1}. Tafel slopes were derived from the corresponding LSV curves. LSV data were recorded with automatic iR compensation, whereas chronoamperometry measurements were performed without iR correction. All measured potentials referenced to Hg/HgO were converted to the reversible hydrogen electrode (RHE) scale according to the following equation:

$$E_{RHE} = E_{Hg/HgO} + 0.0591 \times pH + 0.098\ V$$

Ionic Conductivity Measurement

The ionic conductivity of the electrolytes (1.0 M KOH containing varying concentrations of glycerol) was measured using a commercial benchtop conductivity meter (Mettler Toledo SevenExcellence S470-Kit) equipped with an InLab 731-ISM electrode. Prior to measurement, the instrument was calibrated with a standard aqueous KCl solution (111.8 mS cm^{-1}). All measurements were conducted under ambient conditions at 23 ± 2 °C. For each measurement, the probe was fully immersed in freshly prepared electrolyte, and the conductivity was recorded after signal stabilization. Each sample was measured in triplicate, and the average value was reported to ensure accuracy and reproducibility.

***In situ* Attenuated Total Reflectance Fourier Transform Infrared (ATR-FTIR) Measurement**

In situ ATR-FTIR spectra were recorded on a Bruker INVENIO S spectrometer equipped with an MCT detector and a Si ATR crystal, over the spectral range of 4000–1000 cm^{-1} with a resolution of 4 cm^{-1}. Electrochemical measurements were controlled by a CHI 760E electrochemical workstation using a commercial FTIR spectroelectrochemical cell configured in a three-electrode system. A Hg/HgO (1 M KOH) electrode, a Pt wire, and $Cu_3Mo_2O_9$/NF were employed as the reference, counter, and working electrodes, respectively. To ensure reliable contact, controlled mechanical pressure was applied to the $Cu_3Mo_2O_9$/NF electrode using a top micrometer assembly. After initial contact, the micrometer was tightened by 1.5–2.0 turns for each measurement, ensuring intimate and reproducible contact with the Si ATR crystal while minimizing the presence of excess bulk electrolyte. The anolyte consisted of either 0.5 M or 6.0 M KOH containing 0.2 M solketal, while the catholyte was 0.5 M or 6.0 M KOH. Time-resolved FTIR spectra were recorded at 1.4 V *versus* RHE over a period of 0 to 30 minutes under chronoamperometric conditions.

Product Analysis

Both the as-synthesized and commercial samples of solketal and glyceric acid were analyzed by ^{1}H nuclear magnetic resonance (^{1}H NMR) spectroscopy (400 MHz). Liquid-phase reaction products obtained after electrolysis were analyzed by high-performance liquid chromatography

(HPLC) and quantified using external standard calibration curves. HPLC analysis was performed on a Shimadzu LC-2050 system equipped with a refractive index detector (RID-20A). The analytical columns included a Bio-Rad Micro-Guard cation H^+ guard cartridge (4.6 × 30 mm), a Bio-Rad Aminex HPX-87H column (7.8 × 300 mm), and a Shodex Sugar SH1011 column (8.0 × 300 mm). All columns in the series were kept at 30 ℃. An aqueous 8 mM H_2SO_4 solution was used as the mobile phase at a flow rate of 0.25 mL min^{-1}. For each HPLC analysis, 500 μL of electrolyte was withdrawn from the electrochemical cell and acidified with H_2SO_4 before analysis. Reaction products were identified by comparing retention times with those of authentic standard solutions, and concentrations were determined from corresponding calibration curves.

i. The product selectivity was calculated using the following equation:

$$\text{Selectivity (\%)} = \frac{C_{\text{prodcut}}}{\sum C_{\text{product}}} \times 100\%$$

ii. The conversion of solketal was determined according to:

$$\text{Conversion (\%)} = \frac{C_{\text{before}}V_1 - C_{\text{after}}V_2}{C_{\text{before}}V_1} \times 100\%$$

Where C_{before} and C_{after} denote the glycerol concentrations before and after electrolysis, respectively, and V_1 and V_2 represent the electrolyte volumes before and after electrolysis, respectively.

iii. The Faradaic efficiency (FE) was calculated based on the electron transfer associated with each product as follows:

$$\text{FE (\%)} = \frac{4C_{\text{glyce}} + \frac{22}{3}C_{\text{oxa}} + \frac{8}{3}C_{\text{form}}}{Q} \times V \times F \times 100\%$$

Where, C_{glyce}, C_{oxa}, and C_{form} are the concentrations (mM) of glycerate, oxalate, and formate, respectively; V is the volume of electrolyte (10 mL); F is the Faraday constant (96485 C mol^{-1}); and Q is the total charge (C) passed during electrolysis.

iv. The carbon balance (CB) was calculated based on the following equation:

$$\text{CB (\%)} = \frac{(C_3\text{product} + \frac{2}{3}C_2\text{product} + \frac{1}{3}C_1\text{product})V_2}{C(\text{GLYi})V_1 - C(\text{GLYf})V_2} \times 100\%$$

Where *C(GLYi)* and *C(GLYf)* represent the initial and final concentrations of glycerol, respectively, and V_1 and V_2 are the corresponding electrolyte volumes before and after the reaction.

Computational methods

All first-principles calculations were performed within the framework of density functional theory (DFT) using the Vienna *ab initio* Simulation Package (VASP).[S1, S2] The projector-augmented

wave (PAW) method was employed to describe electron–ion interactions, with a plane-wave energy cutoff of 450 eV [S3]. The exchange–correlation functional was treated within the generalized gradient approximation (GGA) using the Perdew–Burke–Ernzerhof (PBE) parameterization [S4-S7]. Long-range van der Waals interactions were accounted for using the DFT-D3 correction method with Becke–Johnson damping.

For geometry optimization, the atomic positions of solketal and solketalate were fully relaxed until the forces on all atoms were less than 0.01 eV/Å and the total energy convergence criterion was set to 10^{-5} eV. The Brillouin zone was sampled using a Monkhorst–Pack k-point mesh of 1 × 1 × 1 for structural relaxations and 3 × 3 × 3 for electronic structure calculations. To prevent spurious interactions between periodic images, a vacuum spacing of 15 Å was applied in all three (x, y, and z) directions.

To analyze chemical bonding and electron localization, the crystal orbital Hamilton population (COHP) and electron localization function (ELF) were computed using the Local Orbital Basis Suite Towards Electronic Structure Reconstruction (LOBSTER) code [S8, S9].

Supplementary Figures

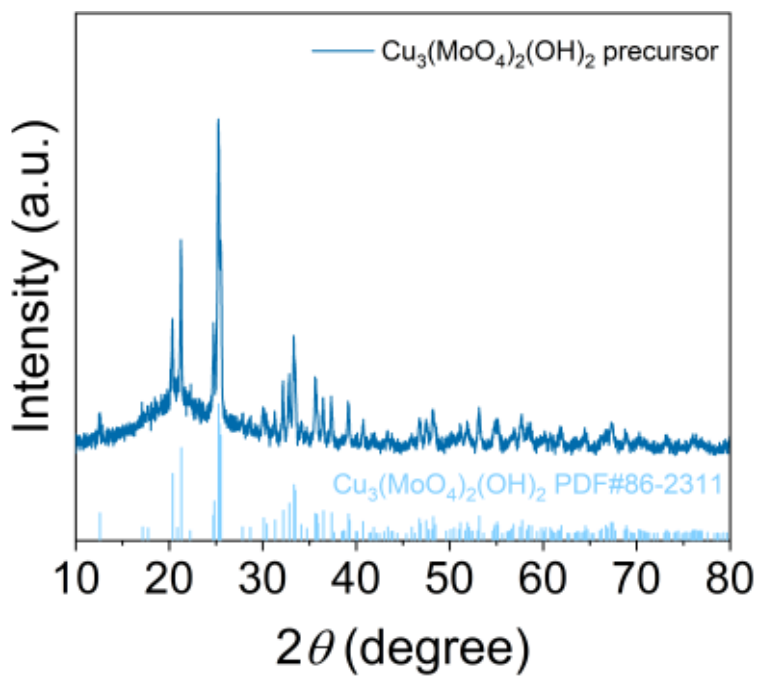

Figure S1. XRD pattern of $Cu_3(MoO_4)_2(OH)_2$.

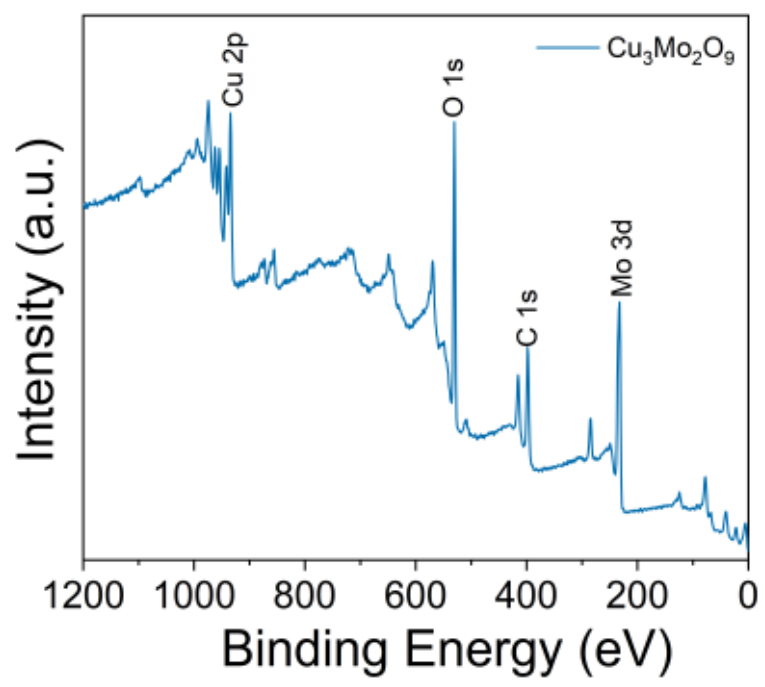

Figure S2. XPS survey spectrum of $Cu_3Mo_2O_9$.

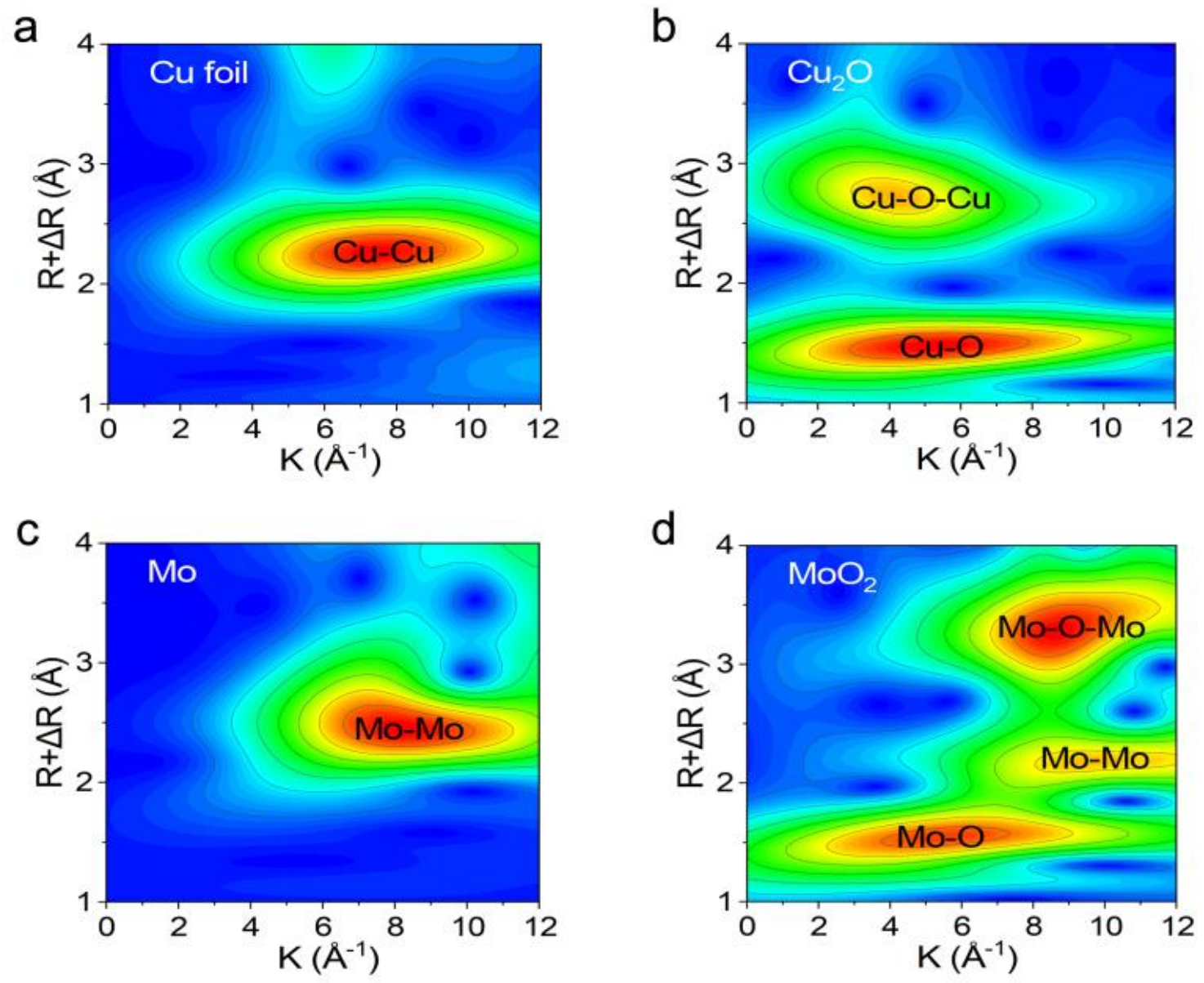

Figure S3. Wavelet transform of the Cu K-edge EXAFS spectra for (a) Cu foil, (b) Cu_2O, (c) Mo foil, and (d) MoO_2 references.

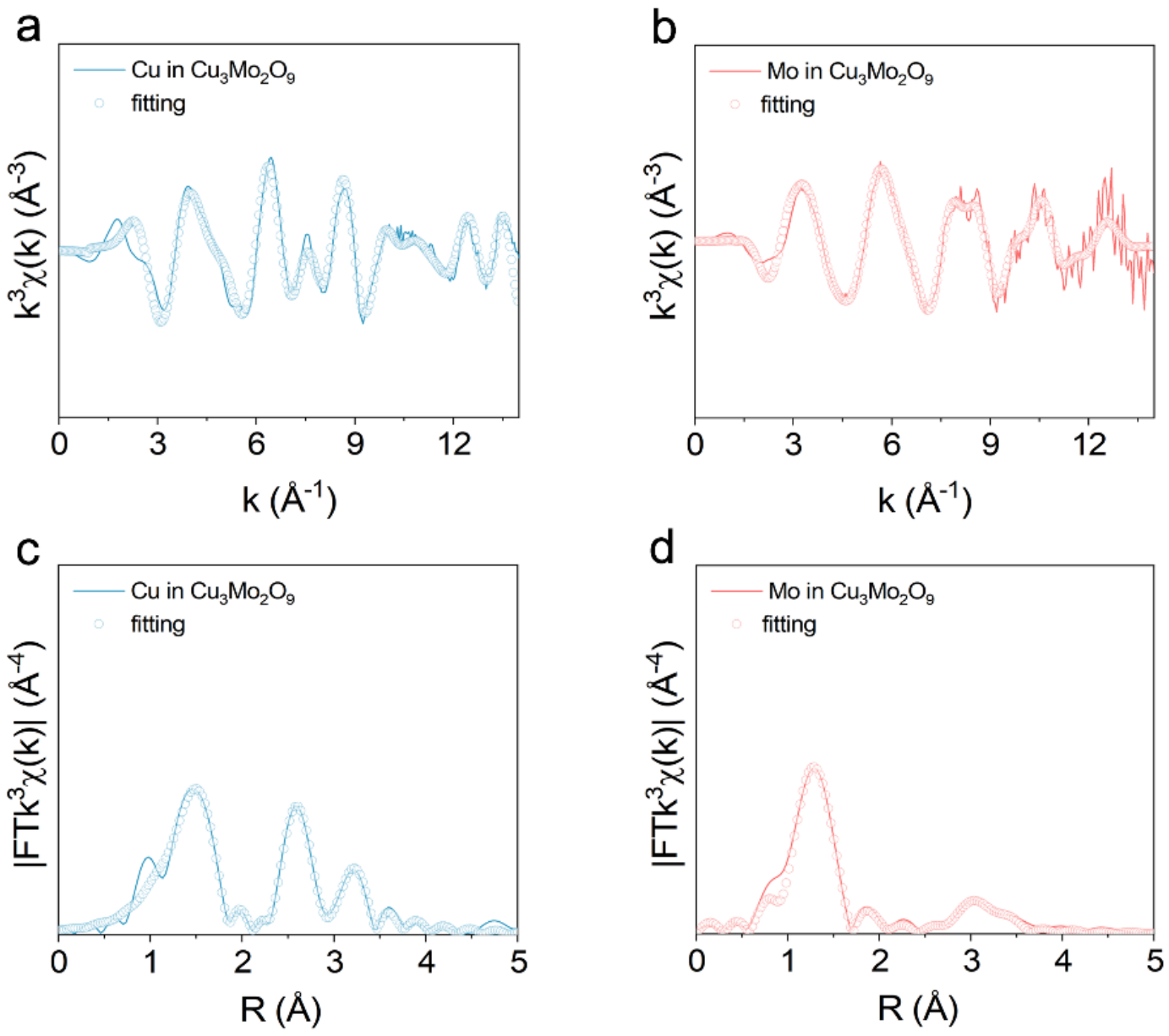

Figure S4. Fitting curves of (a) Cu K-edge and (b) Mo K-edge EXAFS in k-space for $Cu_3Mo_2O_9$. Fitting curves of (c) Cu K-edge and (d) Mo K-edge EXAFS in R-space for $Cu_3Mo_2O_9$.

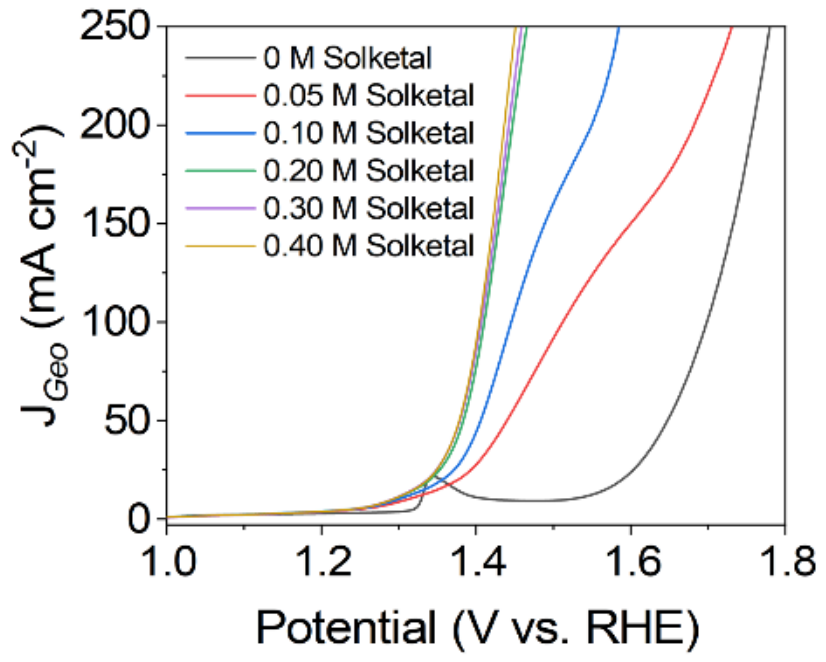

Figure S5. LSV curves of $Cu_3Mo_2O_9$/NF in 1.0 M KOH electrolyte with varying solketal concentrations.

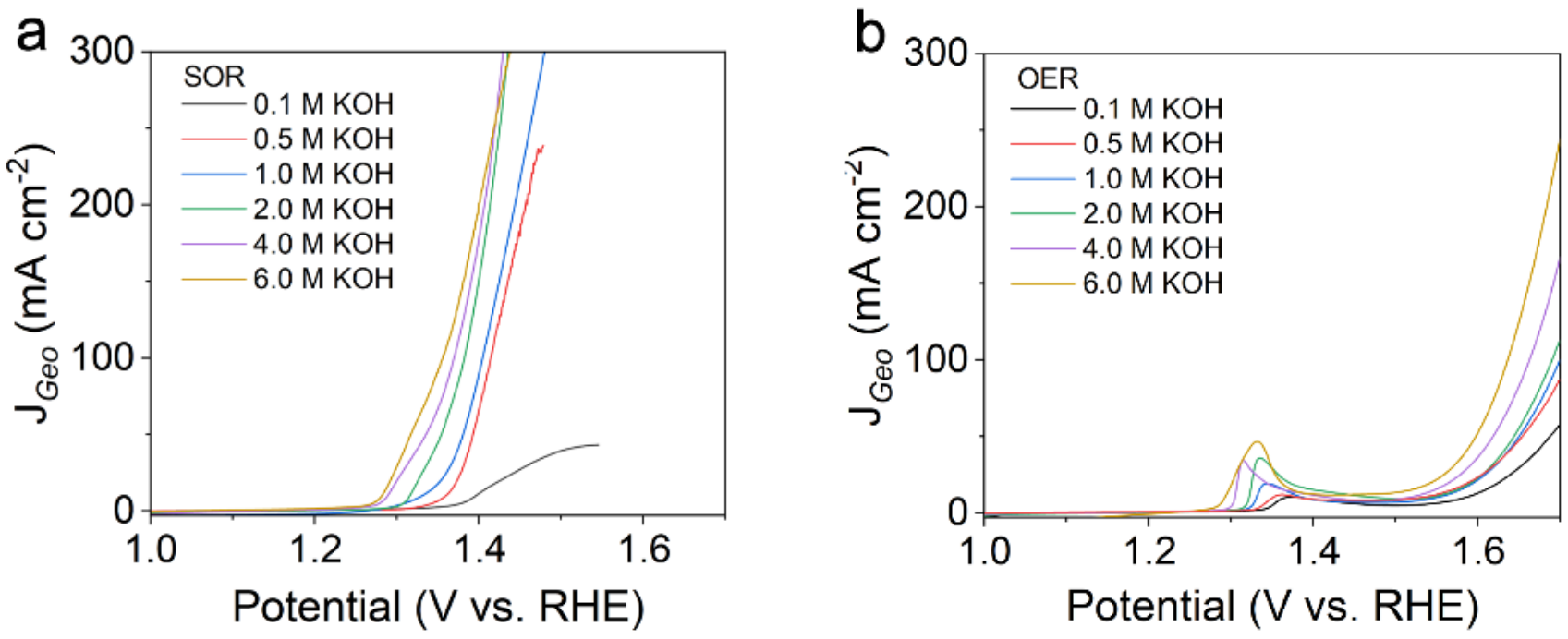

Figure S6. LSV curves of $Cu_3Mo_2O_9$/NF in varying KOH concentrations (a) with and (b) without 0.2 M solketal.

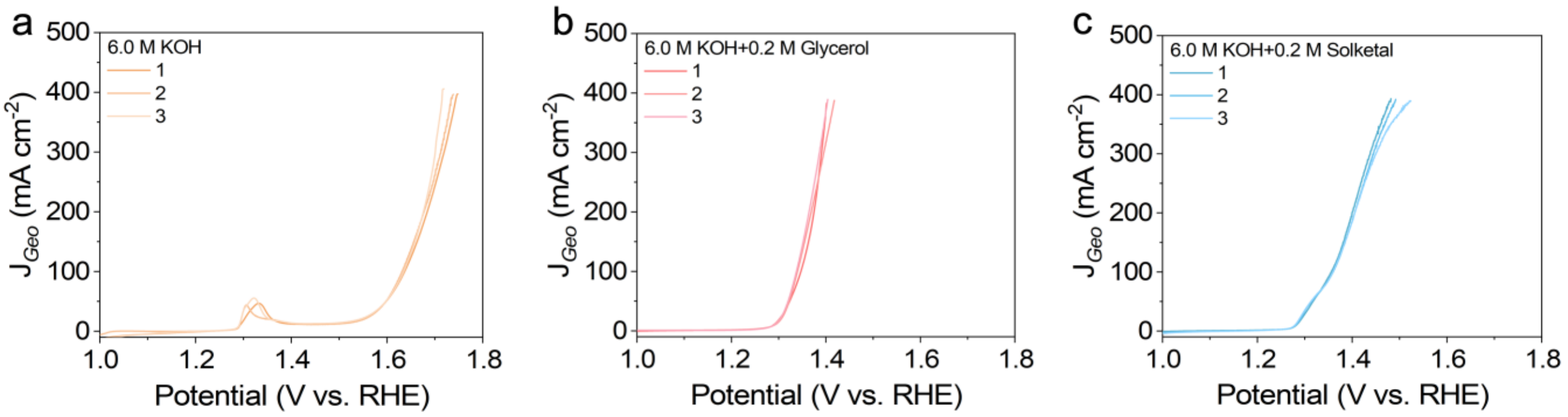

Figure S7. LSV curves of $Cu_3Mo_2O_9$/NF recorded in (a) 6.0 M KOH, (b) 6.0 M KOH with 0.2 M glycerol, and (c) 6.0 M KOH with 0.2 M solketal, based on three independent parallel measurements.

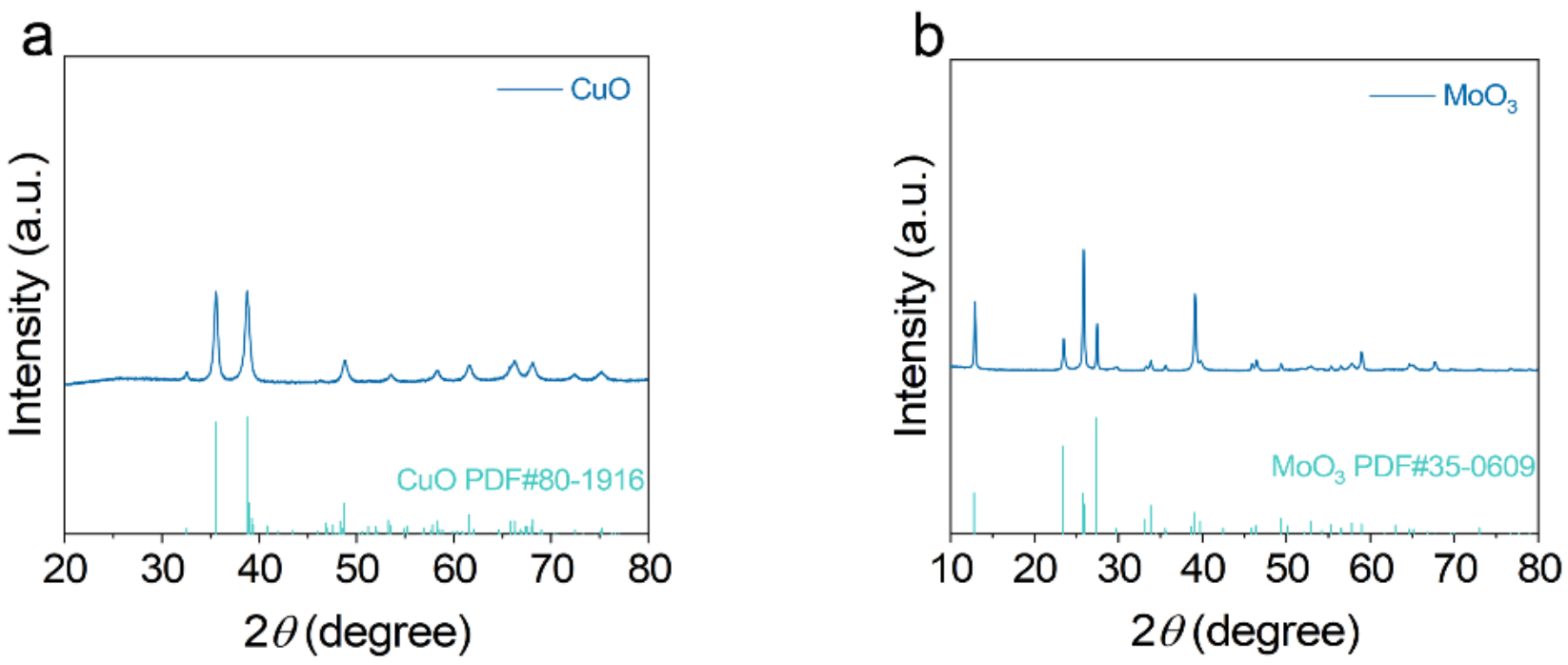

Figure S8. XRD patterns of the synthesized (a) CuO and (b) MoO_3.

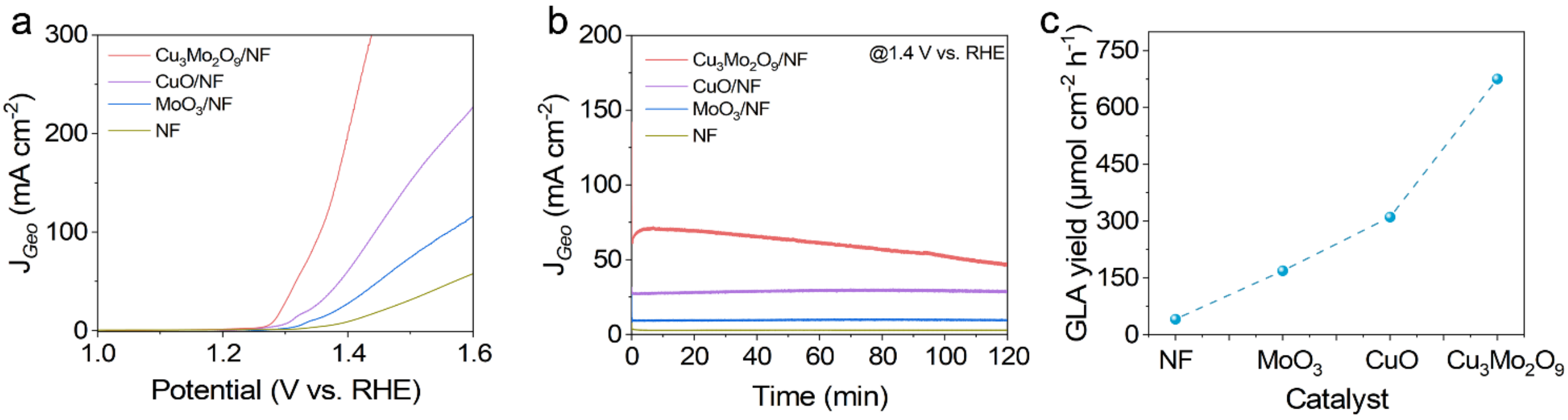

Figure S9. (a) LSV curves of $Cu_3Mo_2O_9$/NF, CuO/NF, MoO_3/NF, and bare NF electrodes in 6.0 M KOH with 0.2 M solketal. (b) I-t curves of various electrodes at 1.40 V *versus* RHE. (c) The corresponding GLA yields at various electrodes.

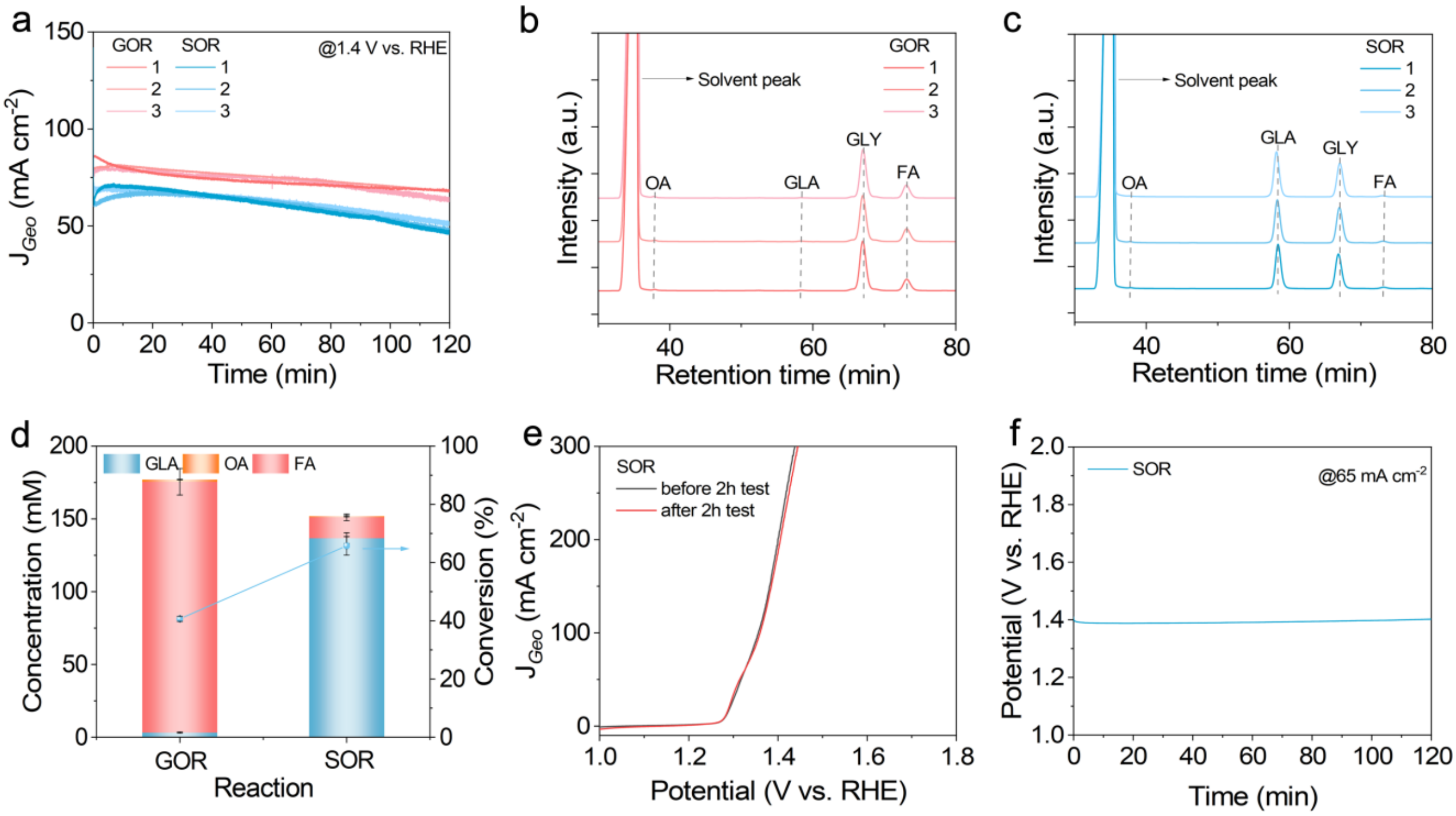

Figure S10. (a) I-t curves of $Cu_3Mo_2O_9$/NF for SOR and GOR at 1.40 V *versus* RHE in 6.0 M KOH with 0.2 M solketal or 0.2 M glycerol, based on three independent parallel experiments. (b) HPLC spectra of products obtained from GOR after electrolysis at 1.40 V *versus* RHE for 2 hours, based on three independent parallel experiments. (c) HPLC spectra of products obtained from SOR after electrolysis at 1.40 V *versus* RHE for 2 hours, based on three independent parallel experiments. (d) Corresponding product concentrations and reactant conversions with error bars derived from three independent parallel experiments. (e) LSV curves of $Cu_3Mo_2O_9$/NF for SOR measured before and after 2 h of chronoamperometric testing in 6.0 M KOH with 0.2 M solketal. (f) Chronopotentiometric curve of $Cu_3Mo_2O_9$/NF for SOR conducted at 65 mA cm^{-2} in 6.0 M KOH with 0.2 M solketal.

Note: The apparent current decay observed in the SOR system during the 2 h of electrolysis (relative to GOR) is a substrate-specific kinetic phenomenon rather than irreversible catalyst degradation (Figure S10a). This behavior is primarily attributed to two factors: (i) Limited electron transfer, as solketal undergoes a single-step partial oxidation, and further electron transfer ceases once it is converted to solketalate; and (ii) Sluggish desorption, where the bulky and rigid isopropylidene group of the solketalate product impedes its release from the surface, leading to temporary site blockage by electrochemically inert species. Importantly, the intrinsic stability of the catalyst is confirmed by the nearly identical LSV curves before and after the 2 h chronoamperometric test (Figure S10e), as well as negligible potential fluctuations (< 0.015 V) during chronopotentiometric testing (Figure S10f).

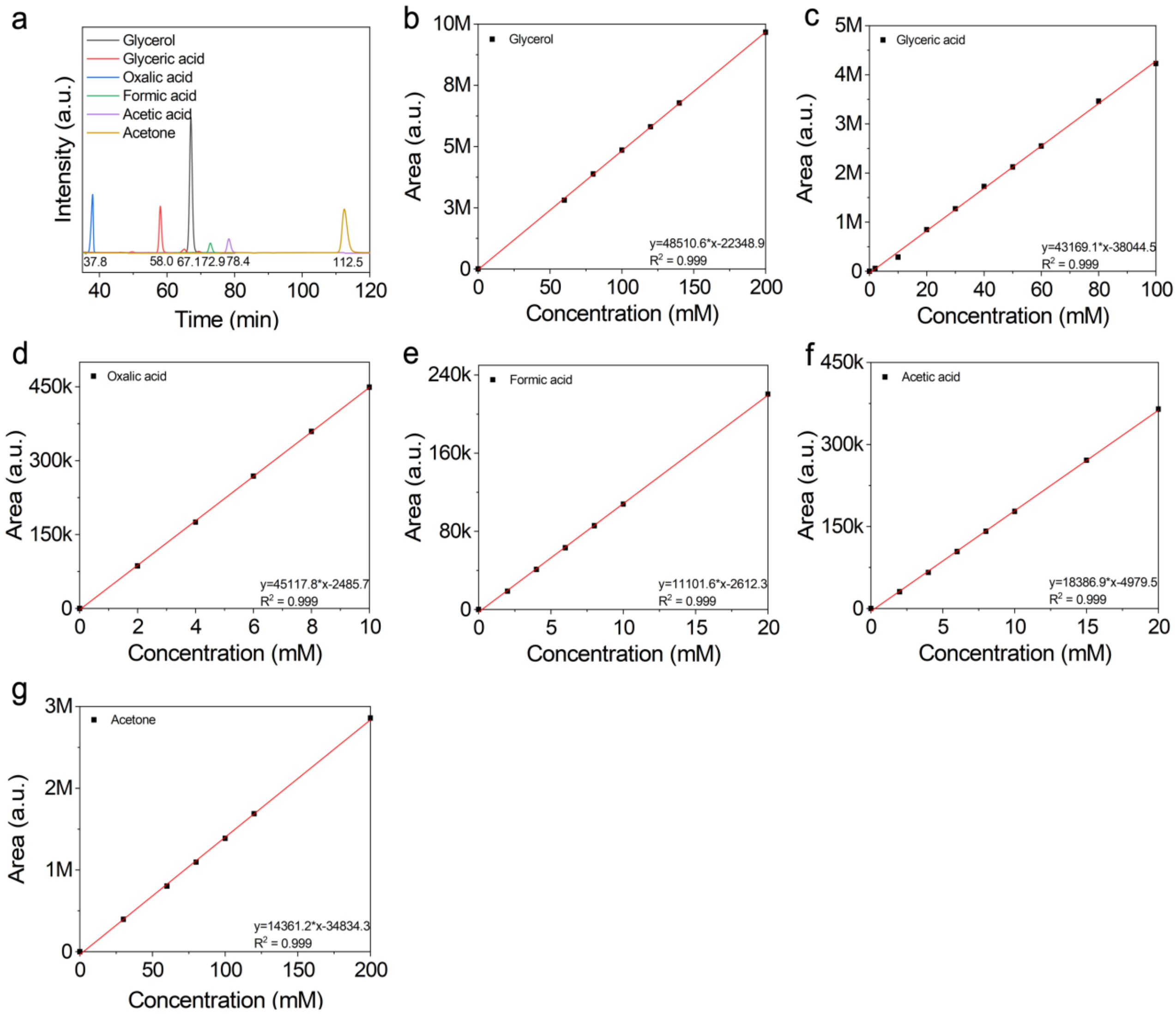

Figure S11. (a) HPLC spectra for the separation of standard products detected by the RID detector. HPLC standard calibration curves for (b) glycerol, (c) glyceric acid, (d) oxalic acid, (e) formic acid, (f) acetic acid, and (g) acetone.

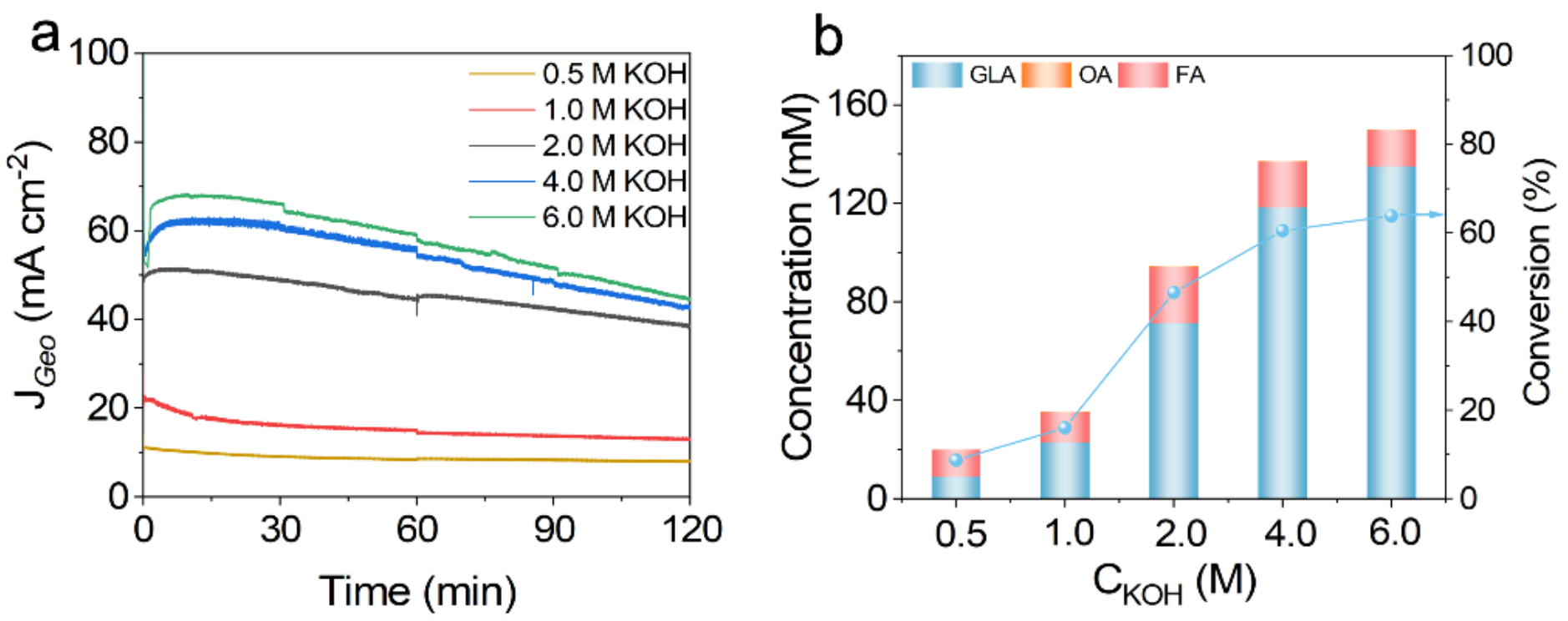

Figure S12. (a) I-t curves of $Cu_3Mo_2O_9$/NF in varying concentrations of KOH electrolyte containing 0.2 M solketal at 1.40 V *versus* RHE. (b) The corresponding product concentration and solketal conversion.

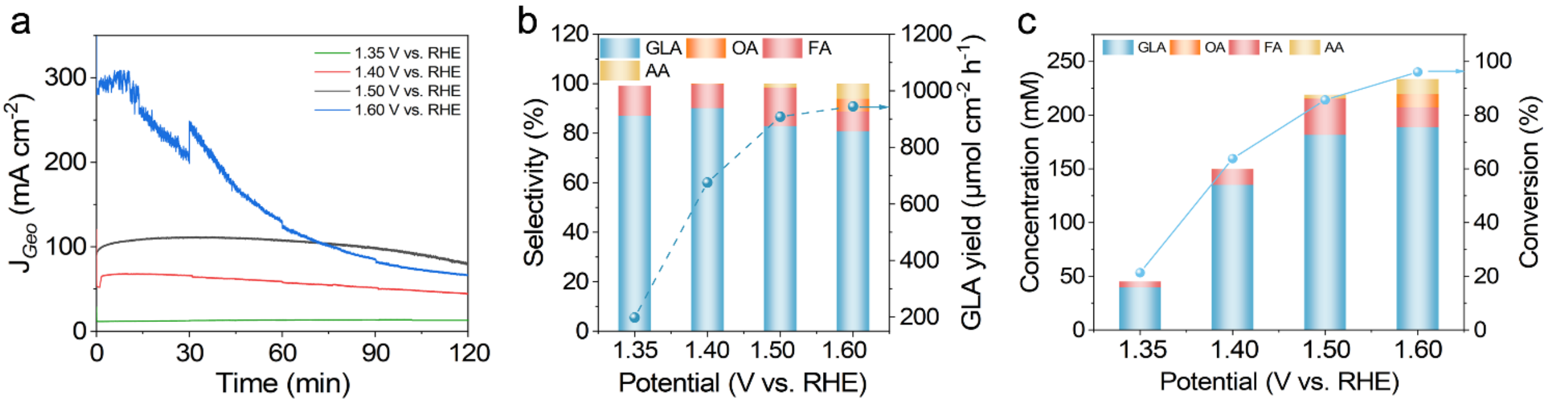

Figure S13. (a) I-t curves of $Cu_3Mo_2O_9$/NF at varying applied potentials in 6.0 M KOH with 0.2 M solketal. (b) Product selectivities and GLA yields at various applied potentials in 6.0 M KOH containing 0.2 M solketal. (c) The corresponding product concentration and solketal conversion.

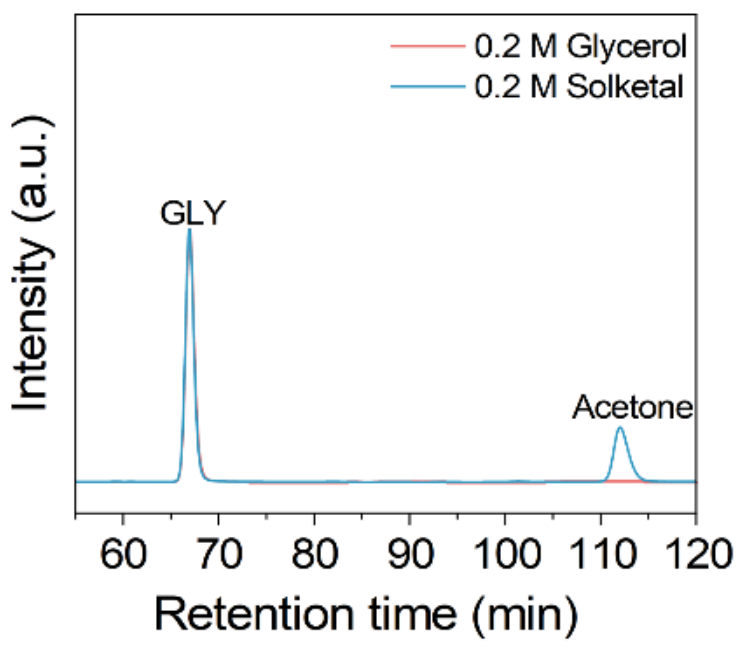

Figure S14. HPLC spectra of 0.2 M solketal and 0.2 M glycerol.

Note: As solketal undergoes rapid and complete hydrolysis to an equivalent amount of glycerol under acidic conditions, the measured glycerol conversion accurately reflects the conversion of solketal.

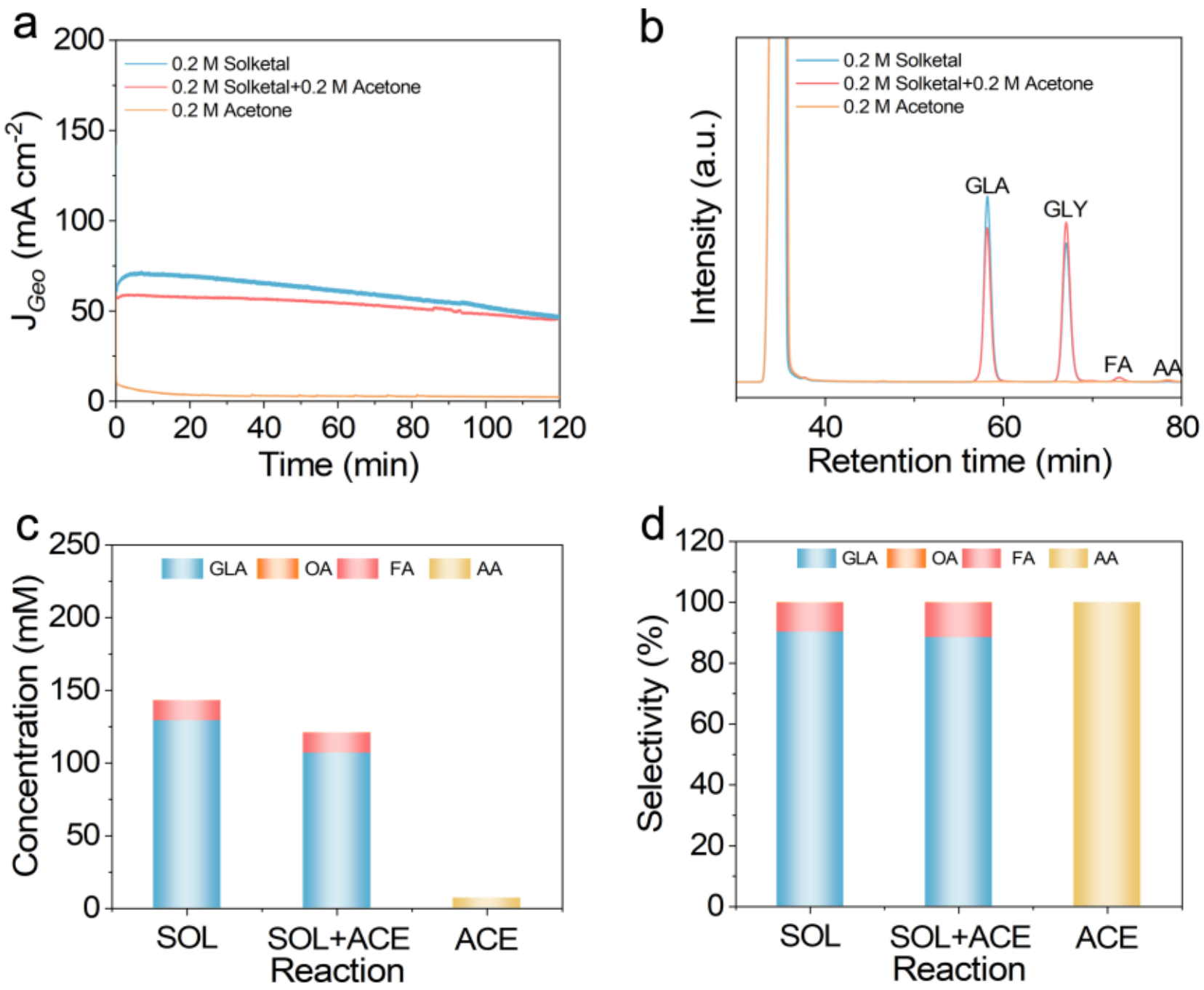

Figure S15. (a) I-t curves of $Cu_3Mo_2O_9$/NF in 6.0 M KOH with 0.2 M solketal, with 0.2 M solketal and 0.2 M acetone, or with 0.2 M acetone at 1.4 V *versus* RHE. (b) HPLC spectra of products obtained after electrolysis at 1.4 V *versus* RHE for 2 hours. (c) The corresponding product concentration. (d) The corresponding product selectivity.

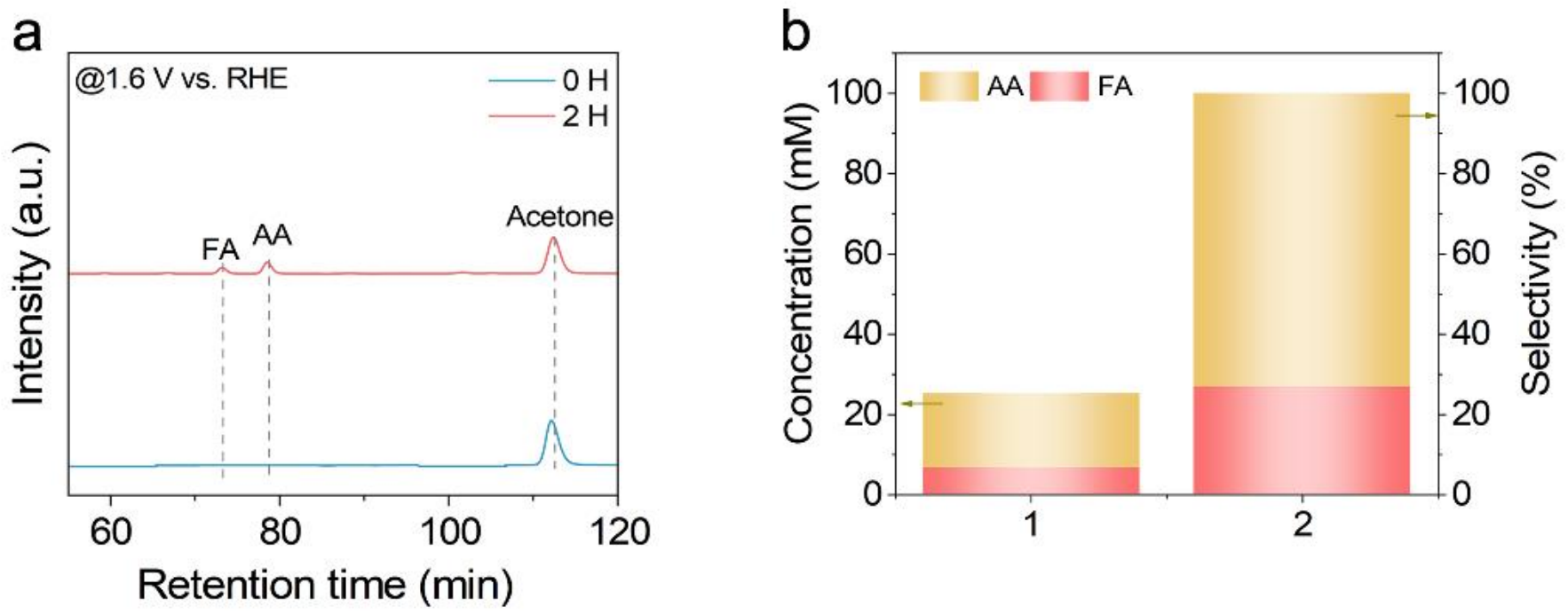

Figure S16. (a) HPLC spectra of the products obtained after electrolysis of 0.2 M acetone in 6.0 M KOH at 1.60 V *versus* RHE for 2 hours. (b) The corresponding product concentration and product selectivity.

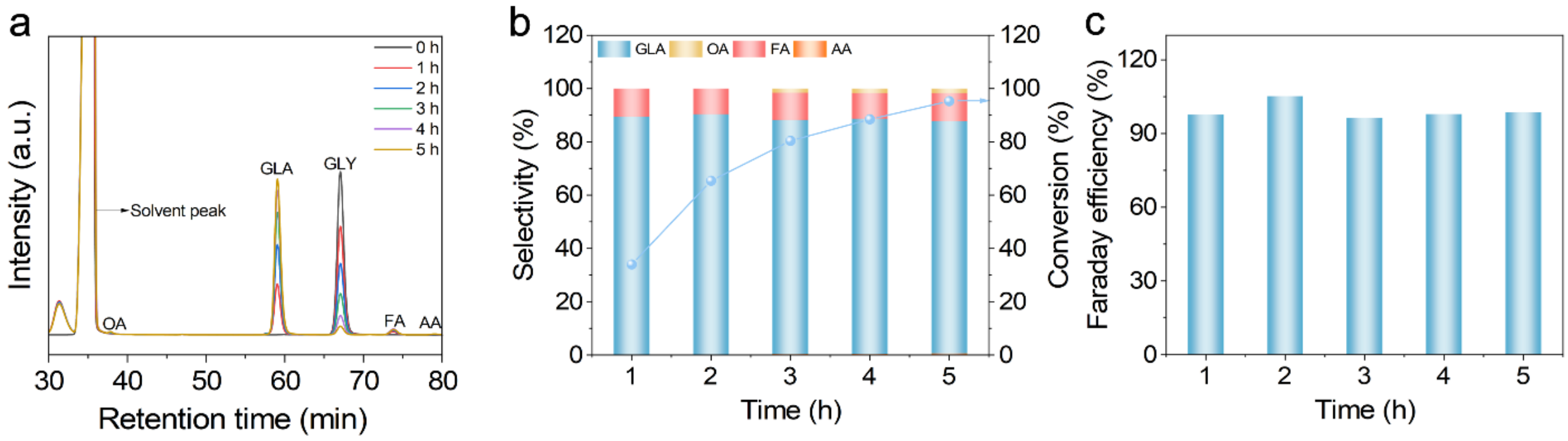

Figure S17. (a) HPLC spectra of products obtained from SOR electrolysis in 6.0 M KOH with 0.2 M solketal at 1.40 V *versus* RHE over 0 to 5 hours. (b) The corresponding product selectivity and conversion over time. (c) The corresponding product Faradaic efficiency over time.

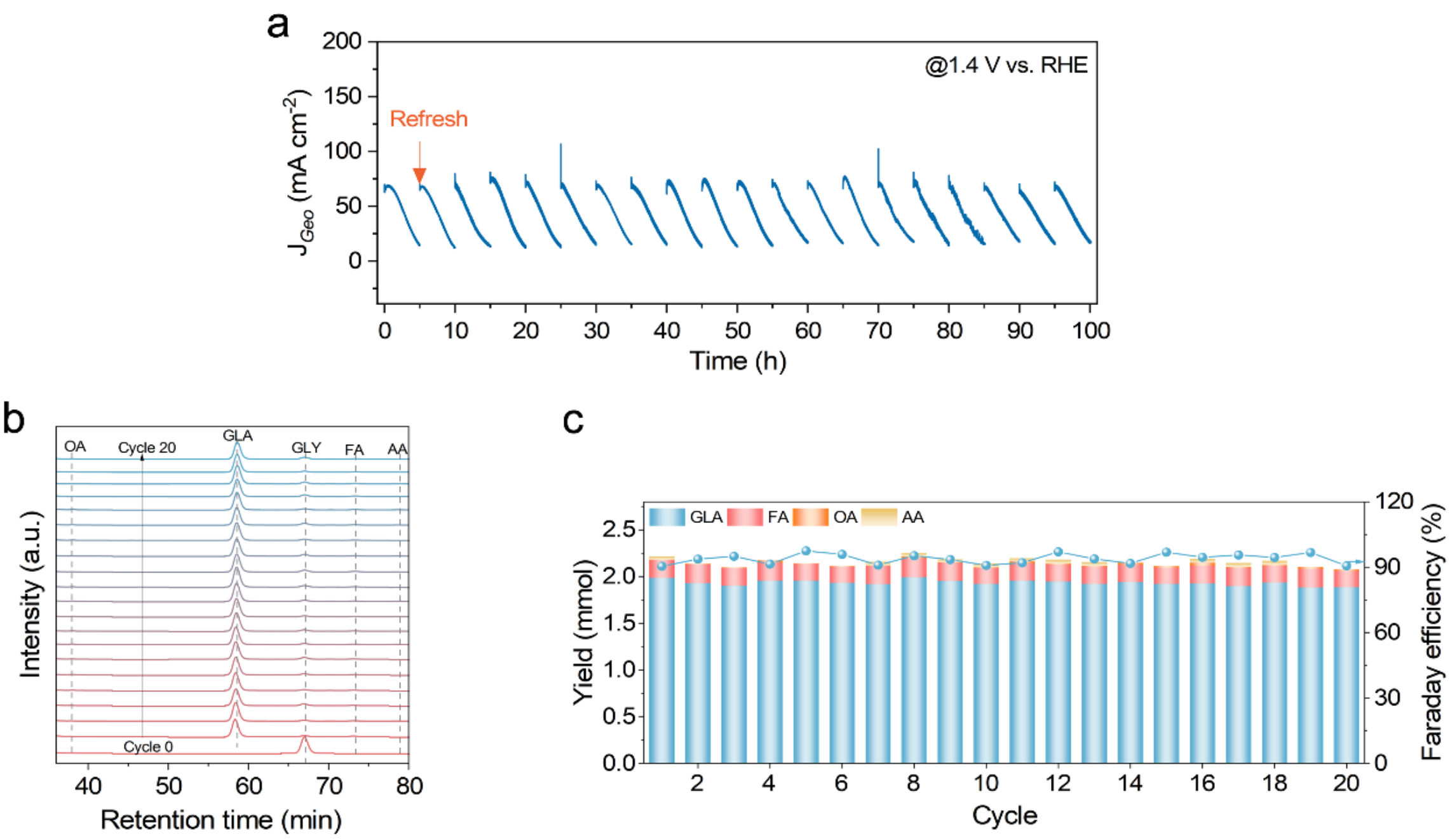

Figure S18. (a) I-t curves of $Cu_3Mo_2O_9$/NF over 20 consecutive electrolysis cycles at 1.40 V *versus* RHE in 6.0 M KOH with 0.2 M solketal. (b) HPLC spectra of products over 20 consecutive electrolysis cycles. (c) The corresponding product yield and Faradaic efficiency of GLA over 20 consecutive electrolysis cycles.

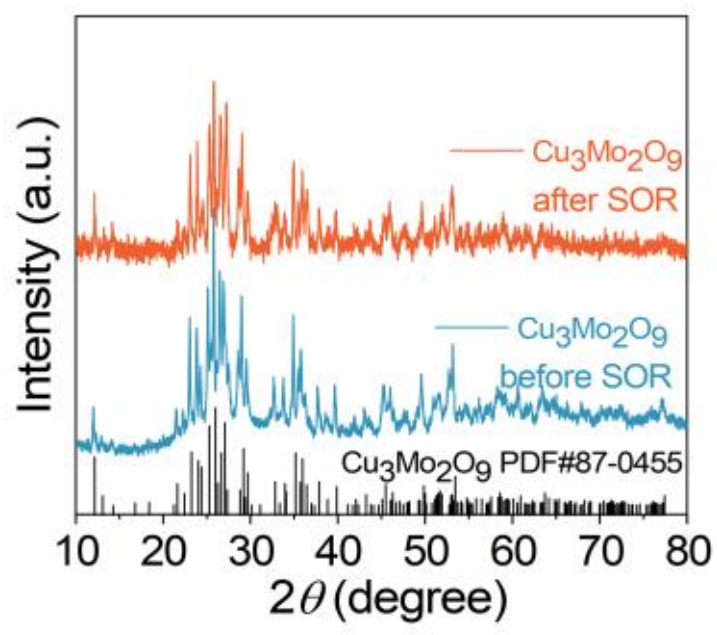

Figure S19. XRD patterns of $Cu_3Mo_2O_9$ before and after 20 consecutive electrolysis cycles.

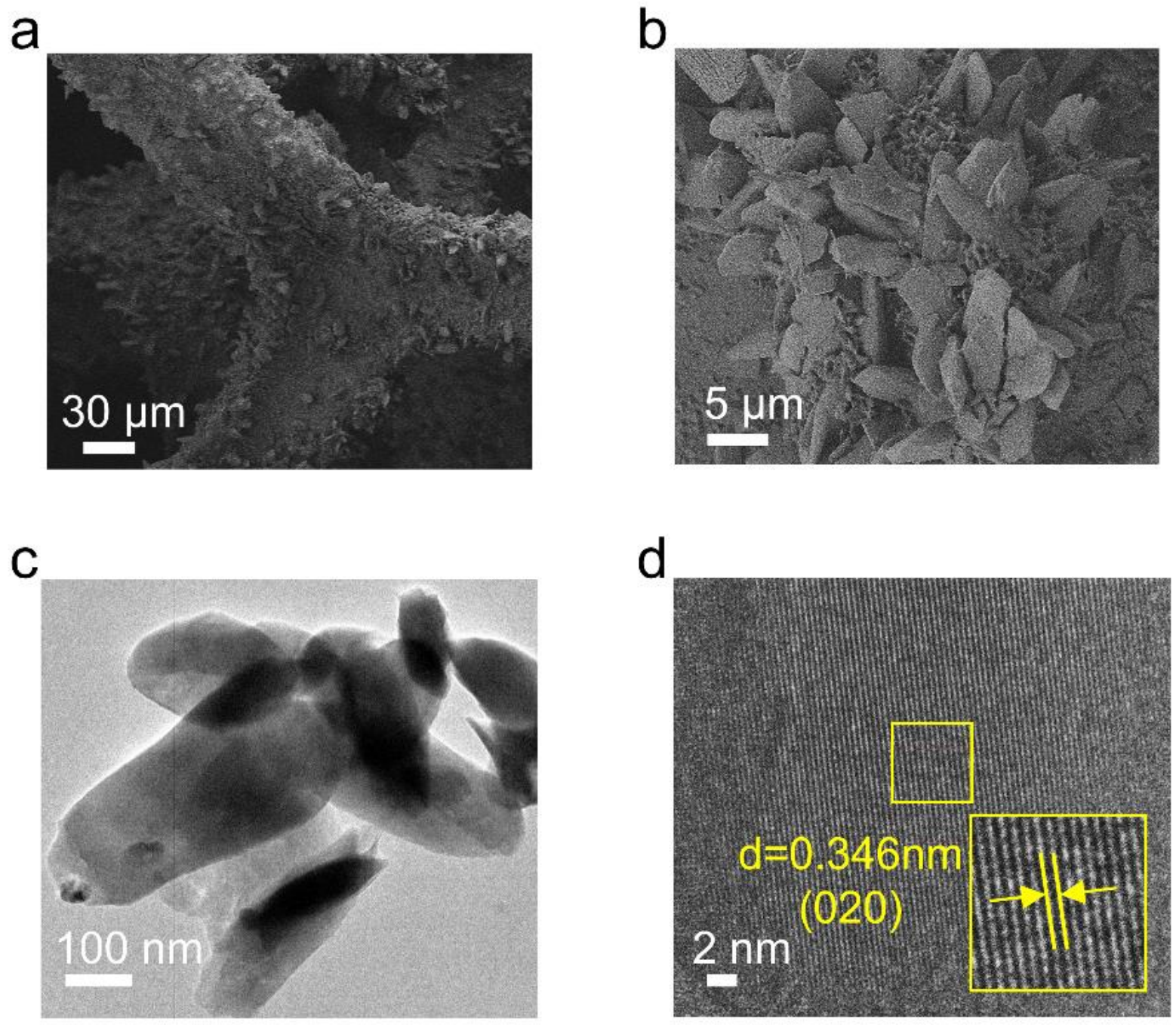

Figure S20. (a, b) SEM images, (c) TEM image, and (d) HRTEM image (inset: enlarged lattice fringes of the selected region) of $Cu_3Mo_2O_9$ after 20 consecutive electrolysis cycles.

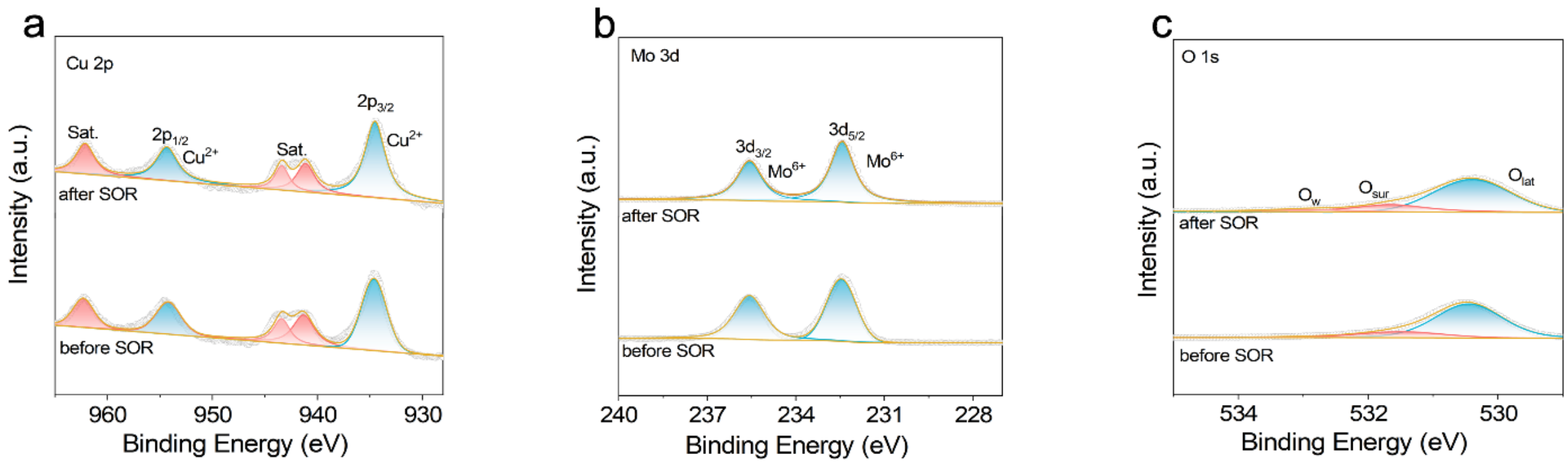

Figure S21. (a) Cu 2p, (b) Mo 3d, and (c) O 1s XPS spectra of $Cu_3Mo_2O_9$ before and after 20 consecutive electrolysis cycles.

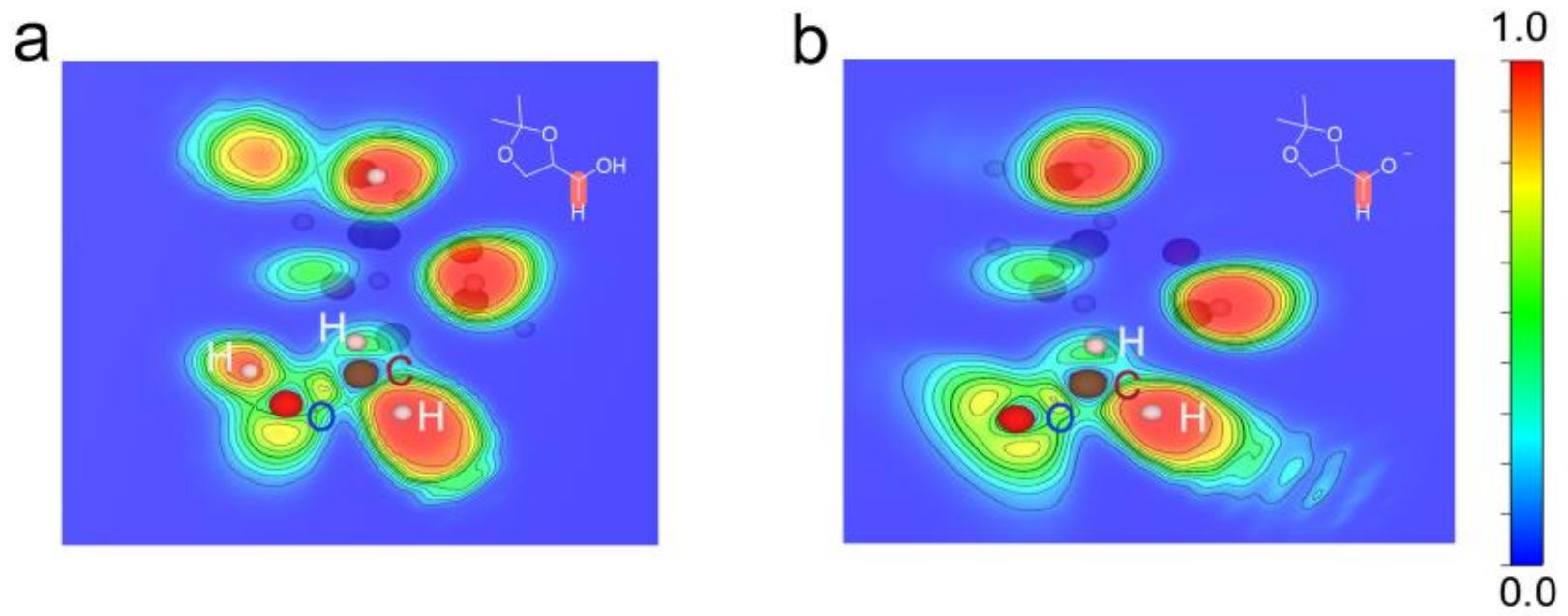

Figure S22. The electron localization function of (a) solketal and (b) its alkoxide form.

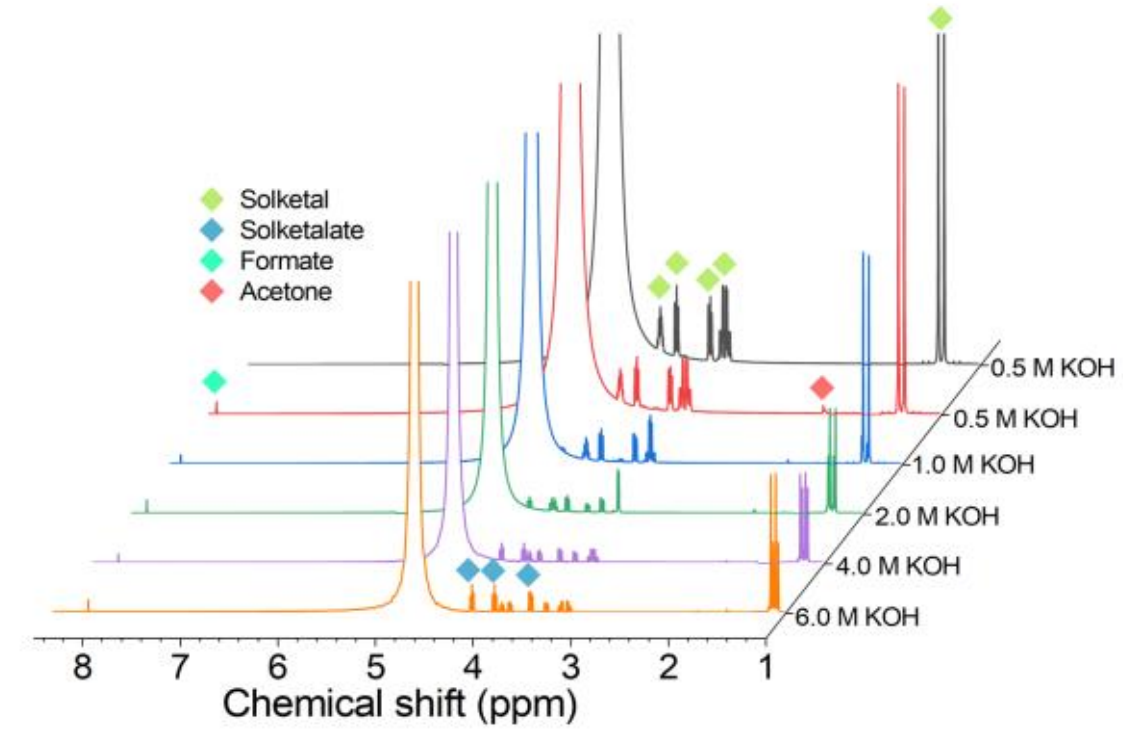

Figure S23. ^{1}H NMR spectra of the electrolyte: before SOR in 0.5 M KOH and after SOR electrolysis at 1.40 V *versus* RHE for 2 hours in different concentrations of KOH.

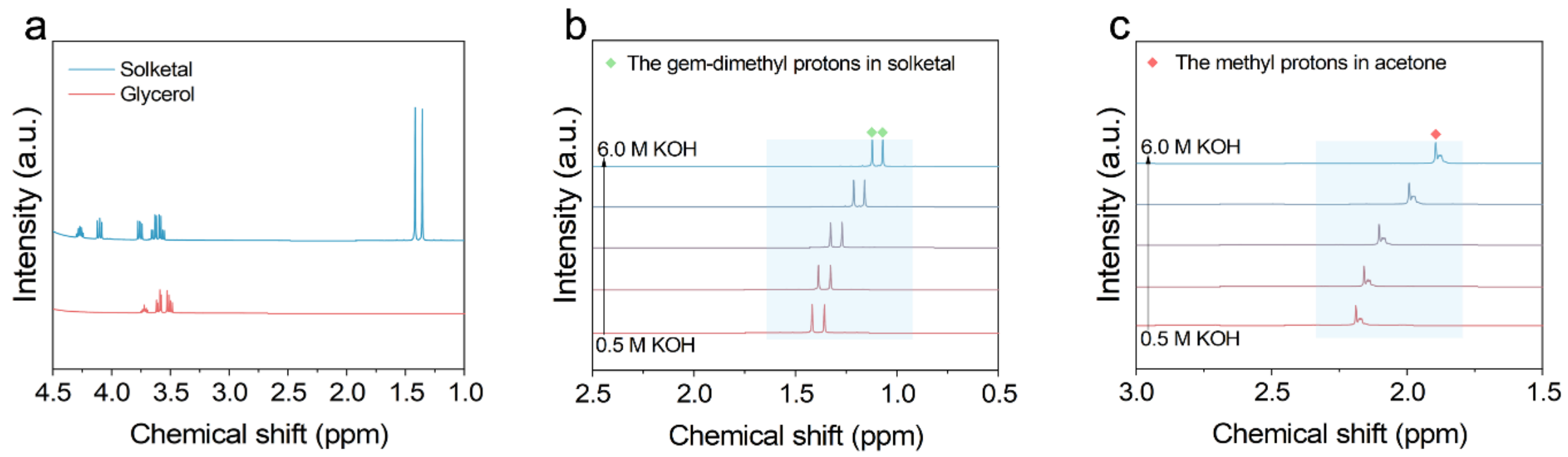

Figure S24. (a) ^{1}H NMR spectra of solketal and glycerol. ^{1}H NMR spectra of (b) the gem-dimethyl protons of solketal and (c) the methyl protons of acetone, recorded in different concentrations of KOH.

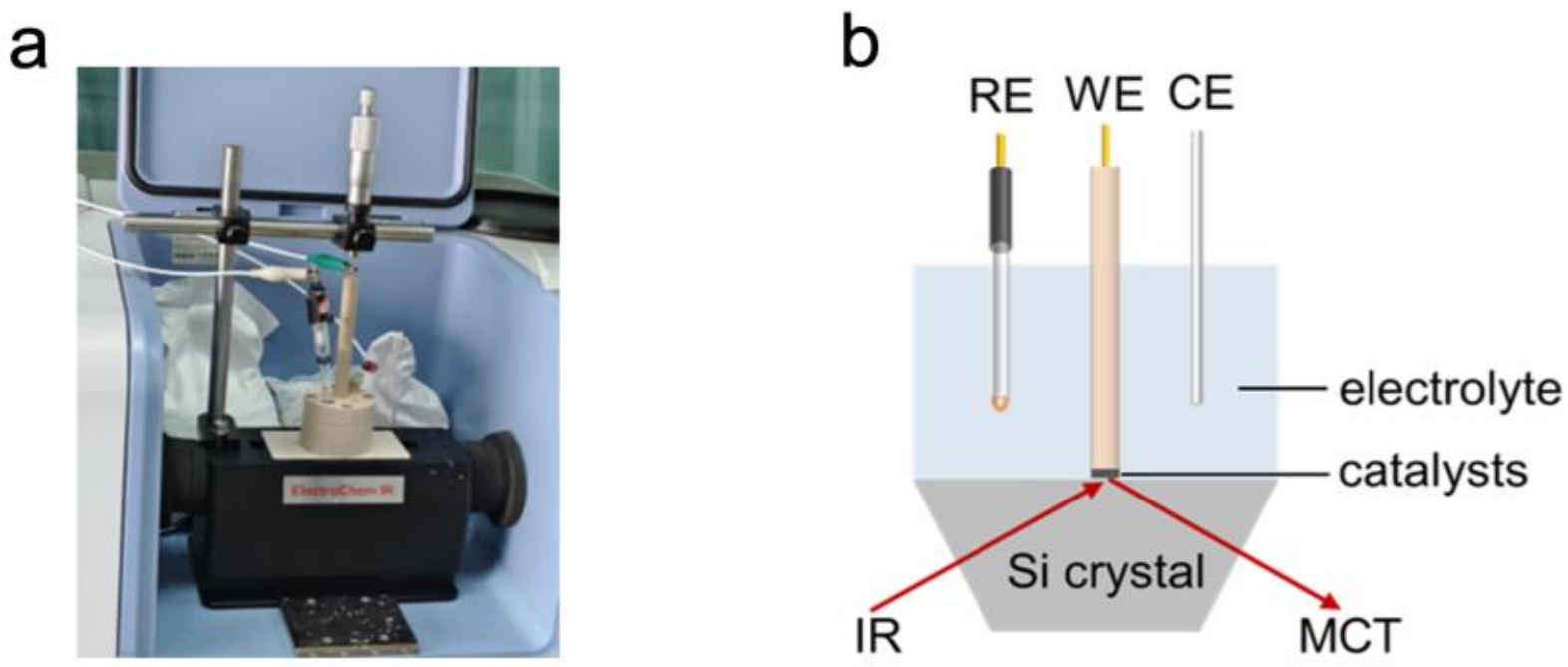

Figure S25. (a) The optical photograph and (b) schematic illustration of the three-electrode electrolytic cell for *in-situ* electrochemical ATR-FTIR measurement.

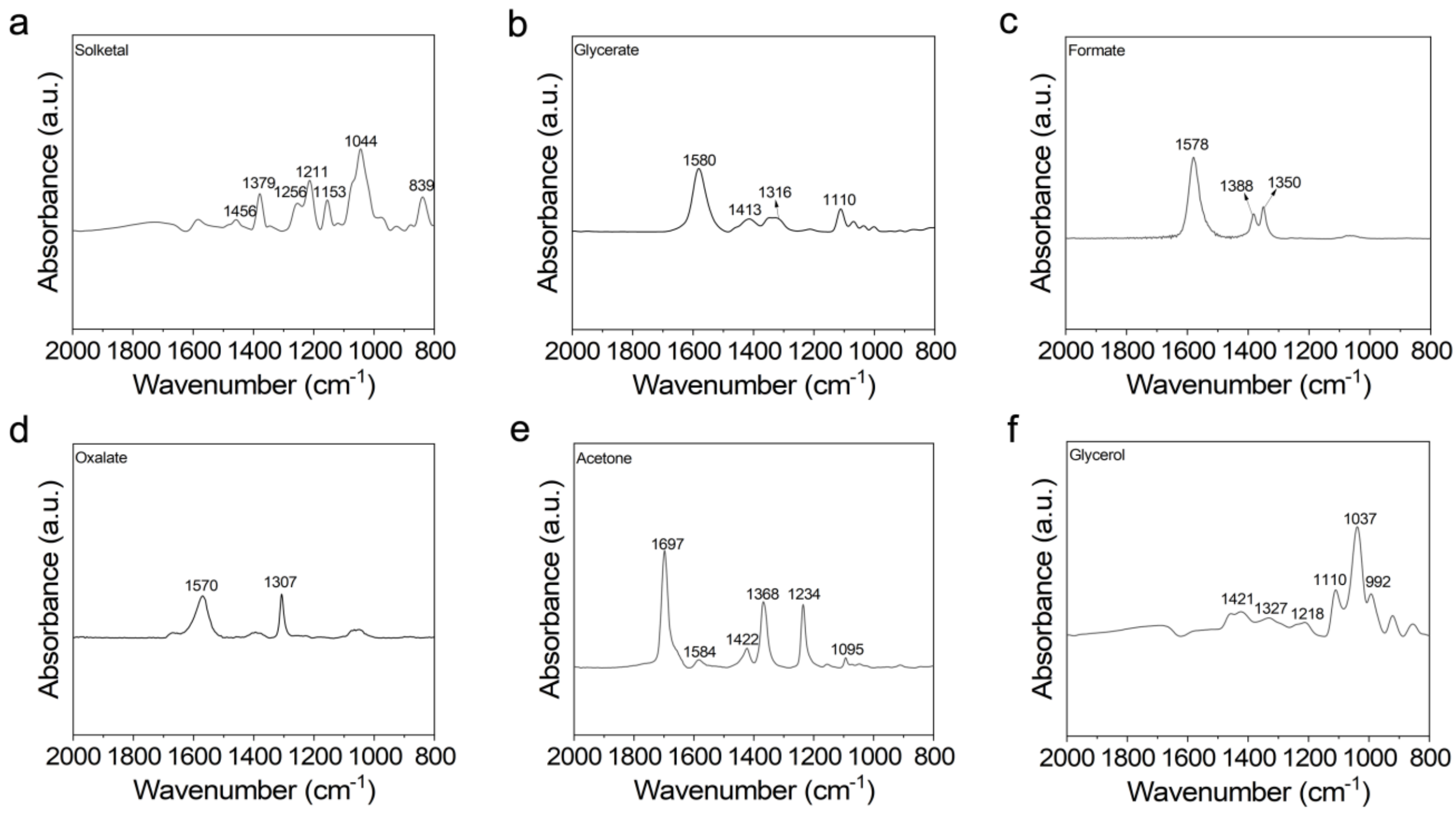

Figure S26. FTIR spectra of (a) solketal, (b) glycerate, (c) formate, (d) oxalate, (e) acetone, and (f) glycerol in KOH solution.

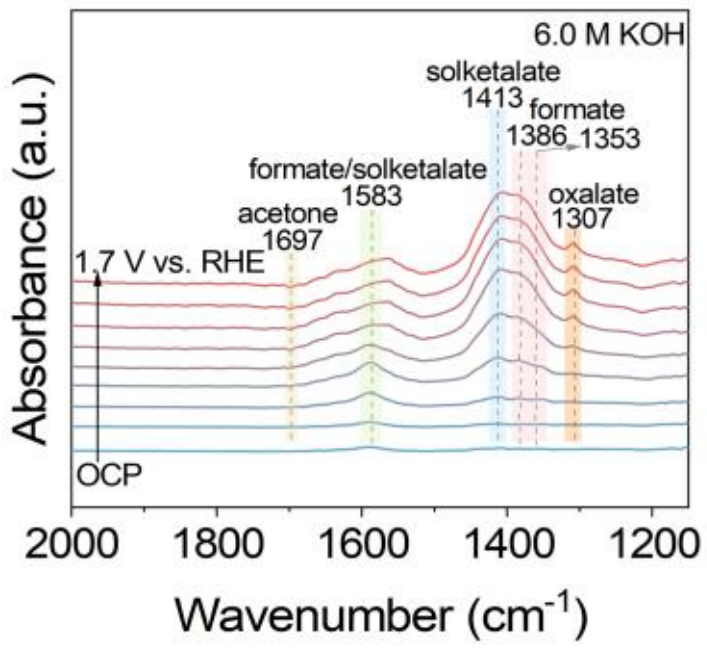

Figure S27. *In situ* ATR-FTIR spectra of $Cu_3Mo_2O_9$/NF at different applied potentials in 6.0 M KOH electrolyte containing 0.2 M solketal.

Note: *In situ* ATR-FTIR measurements recorded on the catalyst from open-circuit potential (OCP) to 1.7 V *versus* RHE in 6.0 M KOH with 0.2 M solketal further demonstrate that solketalate remains the dominant product across the entire potential range. The simultaneous growth of FA and OA signatures (~1307 cm^{-1}) is attributed to C–C bond cleavage promoted at higher anodic potentials. These observations confirm that a highly alkaline environment (6.0 M KOH) effectively suppresses solketal hydrolysis while enhancing catalytic activity and selectivity toward solketalate formation.

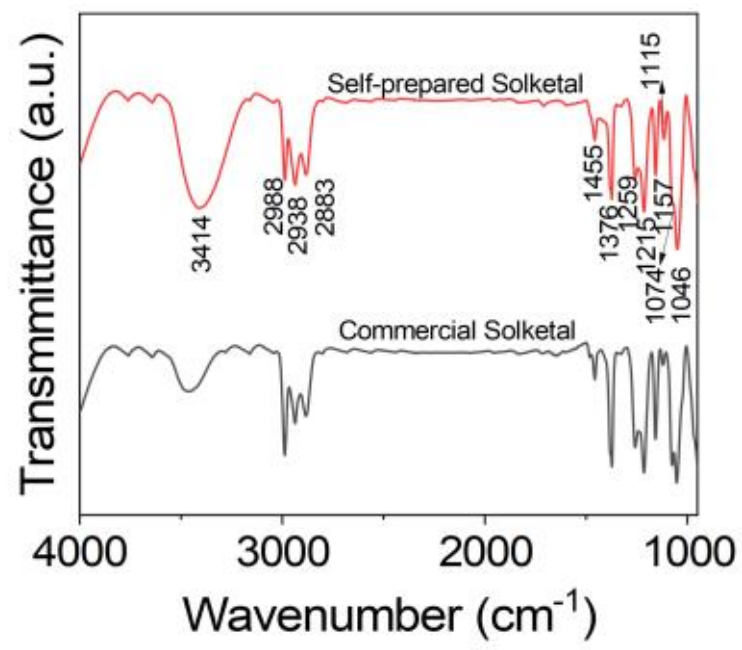

Figure S28. FTIR spectra of self-prepared and commercial solketal.

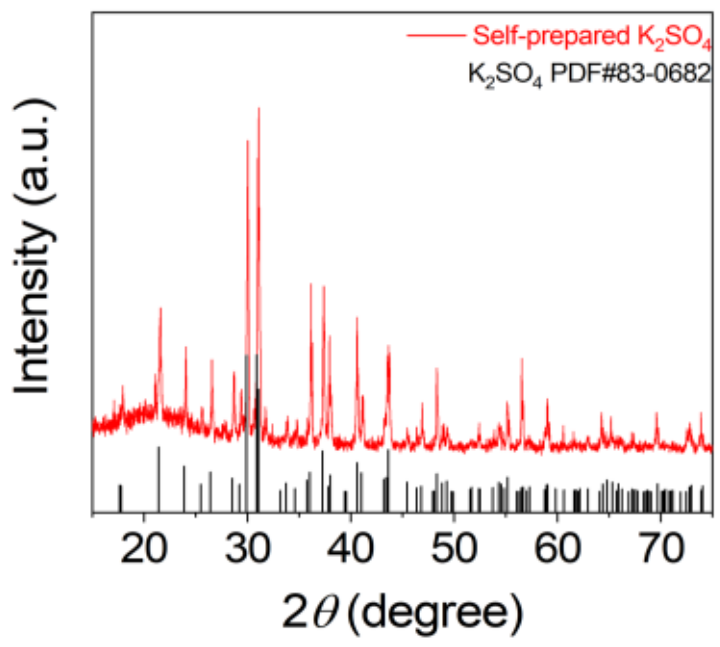

Figure S29. XRD pattern of self-prepared K_2SO_4.

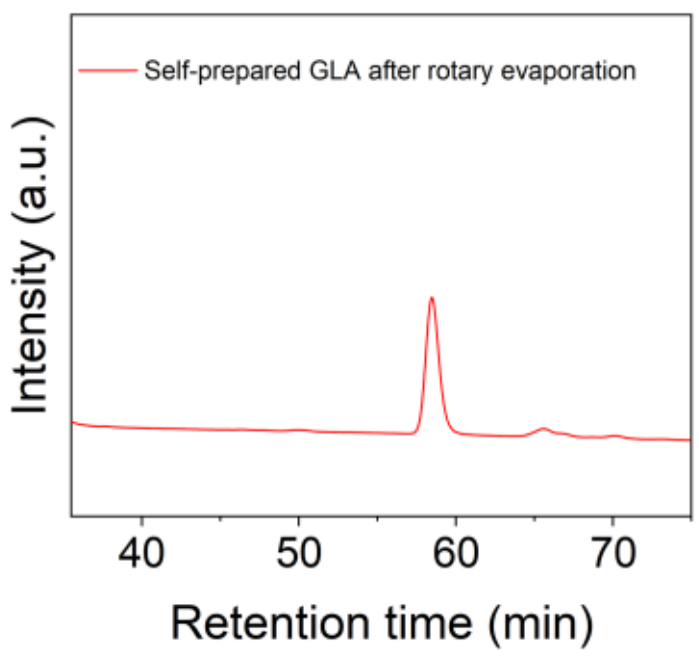

Figure S30. HPLC spectra of self-prepared GLA after reduced-pressure rotary evaporation.

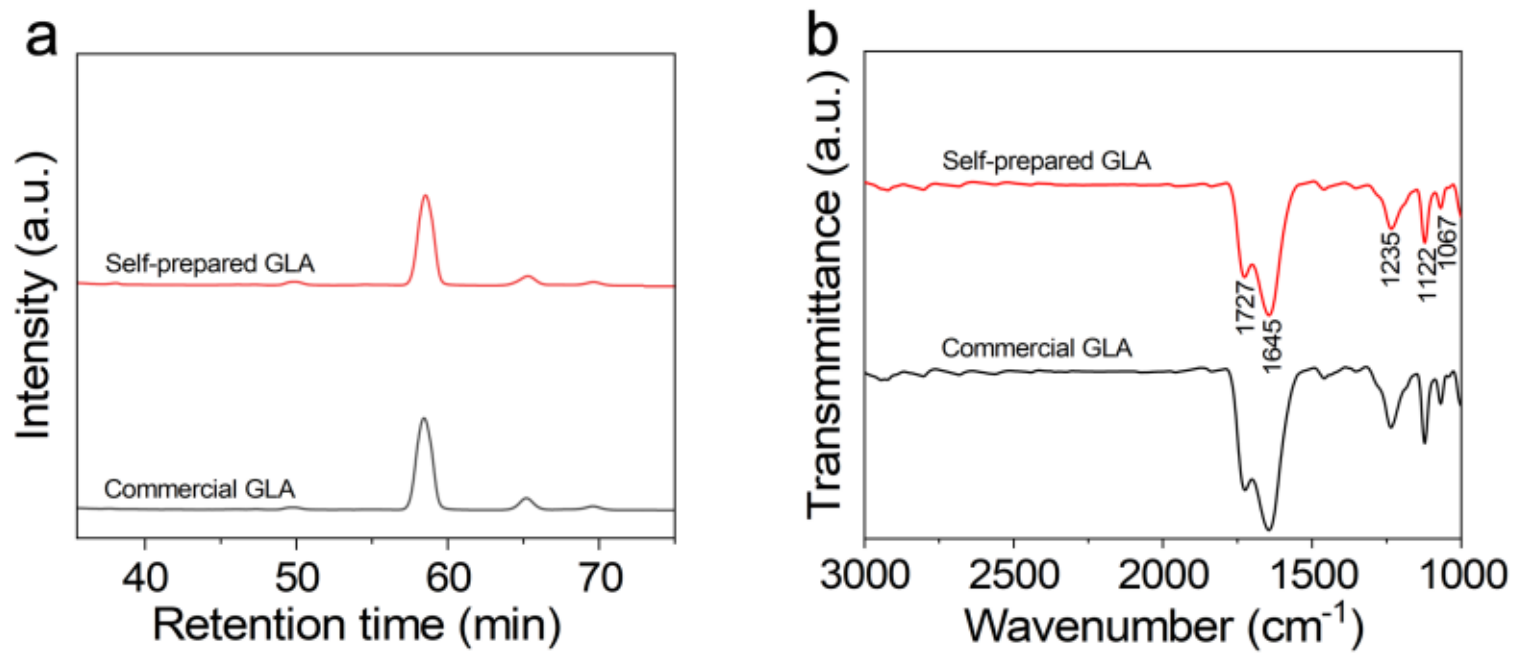

Figure S31. (a) HPLC spectra and (b) FTIR spectra of self-prepared and commercial GLA (20 wt% solution).

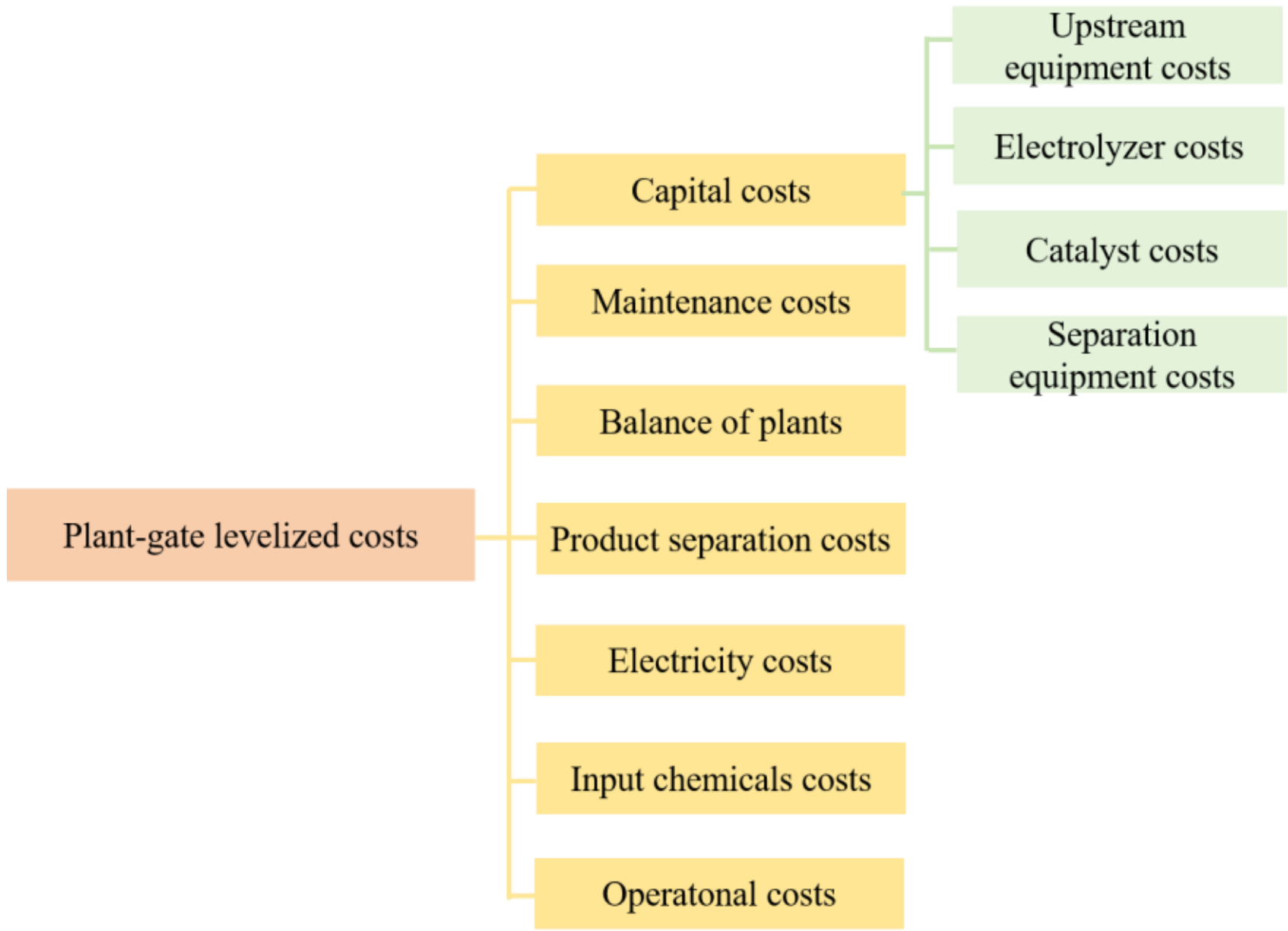

Figure S32. The techno-economic analysis (TEA) model used to evaluate the sustainable electrosynthesis of GLA.

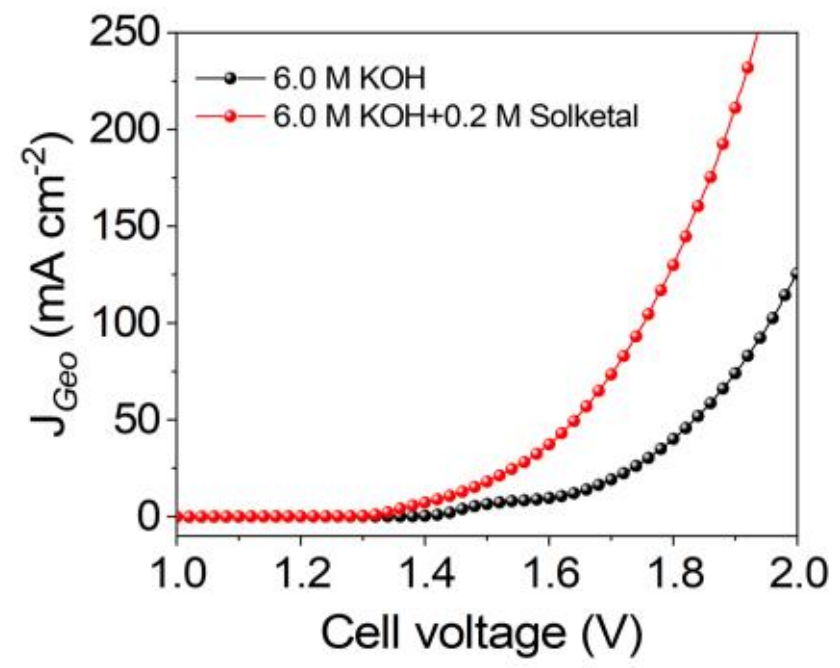

Figure S33. LSV profiles of $Cu_3Mo_2O_9$/NF in 6.0 M KOH with and without 0.2 M solketal using a two-electrode alkaline electrolyzer.

Supplementary Notes

Techno-economic analysis (TEA)

To evaluate the economic potential of renewable electricity-powered GLA production from glycerol and acetone feedstocks, a techno-economic analysis (TEA) was performed. The analytical framework, illustrated in **Figure S32** was used to calculate the total plant-gate levelized cost of production, expressed in US dollars per ton of GLA. The modeled facility was assumed to operate at a processing capacity of 220 tons of glycerol per day. Prices of input chemicals and products employed in the analysis are summarized in Table S6.

The following assumptions were employed in the TEA:

1. The electrolyzer capital cost is assumed to be $10,000 per m^2, and the plant lifetime is set to 10 years. The electrocatalysts' cost is estimated to be 5% of the electrolyzer capital cost.
2. The total capital cost encompasses the entire production pipeline, including upstream equipment for solketal synthesis and downstream acetone recovery (e.g., reactors, distillation columns, and heat exchangers), as well as the initial charge of Amberlyst-15 solid acid catalyst (109.89 tons), the electrolyzer system, electrocatalysts, and product separation equipment. Specifically, the capital cost of the separation equipment is assumed to account for 10% of the electrolyzer capital cost.
3. The electricity price is assumed to be 0.1 $/kWh.
4. The separation cost comprises two main components: energy and material expenditures. The energy consumption for separation is estimated to be 30% of the total electricity cost. Material costs include the single-pass consumption of dilute HCl (no recovery) for back-extraction, as well as solvent make-up costs for the organic extraction phase (n-hexane, n-octanol, trioctylmethylammonium chloride (TOMAC), and 2-naphthaleneboronic acid). A solvent recovery rate of 99% is assumed for the organic phase.

   In practice, separation efficiency and overall process economics depend strongly on the extraction configuration (e.g., single-stage *vs.* multi-stage counter-current extraction) and the separation coefficient. A higher separation coefficient reduces both the solvent-to-feed ratio and the number of stages, thereby lowering both capital and operating costs. While single-stage extraction is operationally simple, it suffers from low efficiency and high solvent consumption. In contrast, multi-stage counter-current extraction enables higher product purity and reduced solvent usage, albeit at increased capital cost. With an optimized multi-stage design, high solvent recovery (~99%) is achievable.
5. The balance of plant cost is set to 58% of the total capital costs.

6. The capacity factor is assumed to be 0.8, corresponding to 19.2 hours of operation per day.

7. It is assumed that 60% of K_2SO_4 and 40% of acetone are recovered and sold as byproducts. The Faradaic efficiency and selectivity toward GLA are both assumed to be 90%. The total cell operating voltage is set to 1.75 V, and the operating current density is 100 mA cm^{-2} (Figure S33).

8. The plant is assumed to process 220 tons of glycerol and 416.66 tons of acetone per day. A glycerol-to-acetone molar ratio of 1:3 is utilized to thermodynamically drive the reversible reaction forward, yielding 284.14 tons of solketal (90% yield). For the subsequent electrooxidation step, a continuous-flow system is assumed. The daily net make-up consumption is modeled using a practical KOH-to-solketal molar ratio of 3:1, corresponding to 362.08 tons of KOH, which is neutralized by 316.46 tons of H_2SO_4. This ratio satisfies the stoichiometric requirement for carboxylate formation, while the excess ensures high ionic conductivity and alkalinity without incurring excessive neutralization costs. Under these optimized continuous-operation conditions, a GLA yield of 90% is achieved, corresponding to a daily production of 205.26 tons.

9. Input chemicals include glycerol, acetone, KOH, and H_2SO_4. The output products include GLA, acetone, hydrogen, and K_2SO_4.

10. The hydrogen selling price is assumed to be $1,900 per ton, with a Faradaic efficiency for hydrogen production of 100%.

11. Both operation and maintenance costs are assumed to be 10% of the total capital costs.

Based on these assumptions, the seven cost and revenue components are calculated on a per-day operating basis.

(1) Material cost

The process requires 220 tons of glycerol, 416.66 tons of acetone, 362.08 tons of KOH, and 316.46 tons of H_2SO_4.

$$\text{Material costs} = \frac{\text{Cost of materials} \times \text{Mass of materials needed}}{\text{mass of GLA produced}}$$

$$= \frac{\$170 \times 220 + 603 \times 416.66 + 820 \times 362.08 + 45 \times 316.46}{205.26} = \$2922.11 \text{ per ton of GLA}$$

(2) Electricity cost

First, the total charge required to oxidize 284.14 tons of solketal per day is calculated as follows:

$$Q = \frac{\text{Mass of solketal converted} \times F \times N}{\text{Molar mass of solketal} \times \text{Faradaic efficiency}}$$

$$= \frac{284.14 \times 10^{6} \times 96485 \times 4}{132.16 \times 0.9} = 9.22 \times 10^{11} \text{C}$$

Where Q is the total charge, F is the Faradaic constant, N = 4 corresponds to the four-electron oxidation of solketal to GLA.

The average current required to sustain this process, assuming a capacity factor of 0.8, is given by:

$$I = \frac{Q}{\text{Time in a day}\times\text{Capacity factor}} = \frac{9.22\times10^{11}\text{C}}{24\times60\times60\times0.8} = 1.33\times10^{7}\text{A}$$

Assuming an operating cell voltage of 1.75 V, the required electrical power is:

$$P = 1.33\times10^{7}\times1.75 = 2.33\times10^{4}\text{kW}$$

The total electrical energy consumption per day is therefore:

$$\text{Energy consumed per day} = \text{P}\times\text{Time in a day}\times\text{Capacity factor}$$

$$= 2.33\times10^{4}\times24\times0.8 = 4.47\times10^{5}\text{kWh}$$

Finally, the daily electricity cost normalized by the mass of GLA produced can be calculated as:

$$\text{Electricity cost per day} = \frac{\text{Energy use per day}\times\text{Cost per kWh}}{\text{Mass of GLA produced}}$$

$$= \frac{4.47\times10^{5}\times0.1}{205.26} = \$217.78 \text{ per ton of GLA}$$

(3) Separation cost

First, the separation energy consumption is assumed to be 30% of the electricity cost :

$$\text{Separation energy cost} = \$217.78\times0.3 = \$65.33 \text{ per ton of GLA}$$

Second, the separation material expenditures account for the continuous recycling of the organic extraction phase, alongside the single-pass consumption of dilute HCl (70.55 tons) used for back-extraction. Assuming an industrial recovery rate of 99% exclusively for the organic phase (meaning only a 1% daily make-up factor is incurred for n-hexane, n-octanol, TOMAC, and 2-naphthaleneboronic acid), the daily material make-up cost is calculated as follows:

$$\text{Separation material cost} = \frac{\text{Cost of separation materials}\times\text{Mass of separation materials needed}}{\text{mass of GLA produced}}$$

$$= \frac{(\$864\times348.5 + 2549\times61.5 + 5564\times1564.1 + 27384\times665.52)\times0.01 + 29.2\times70.55}{205.26}$$

$$= \$1344.2 \text{ per ton of GLA}$$

Finally, the total separation cost is calculated as:

$$\text{Separation cost} = \$65.33 + \$1344.2 = \$1409.53 \text{ per ton of GLA}$$

(4) Capital cost

First, the capital cost for the solketal reaction and downstream acetone recovery equipment (including reactors, distillation columns, and heat exchangers) is estimated based on typical industrial scaling for a ~600 tons per day liquid handling capacity:

$$\text{Cost of solketal equipment}=\$2.5\times10^{6}$$

The capital expenditure for the upstream solketal synthesis is evaluated. The initial load of the Amberlyst-15 solid acid catalyst (109.89 tons) is calculated:

$$\text{Cost of Amberlyst-15 catalyst}=\$2000\times109.89=\$2.2\times10^{5}$$

Second, the electrolyzer area required to sustain the target current is calculated based on an operating current density of 100 mA cm^{-2} (0.1 A cm^{-2}):

$$\text{Area of electrolyzer}=\frac{1.33\times10^{7}}{0.1}=1.33\times10^{8}\ \text{cm}^{2}=1.33\times10^{4}\ \text{m}^{2}$$

The electrolyzer capital cost is assumed to be \$10,000 per m^{2}. Therefore, the total electrolyzer cost is:

$$\text{Cost of electrolyzer}=\$1.33\times10^{4}\times10000=\$1.33\times10^{8}$$

Third, the electrocatalyst cost is assumed to be 5% of the electrolyzer capital cost and is calculated as:

$$\text{Cost of electrocatalyst}=\$1.33\times10^{8}\times0.05=\$6.65\times10^{6}$$

The capital cost of separation equipment is assumed to be 10 % of the electrolyzer capital costs:

$$\text{Cost of separations equipment}=\$1.33\times10^{8}\times0.1=\$1.33\times10^{7}$$

Finally, the annualized capital cost contribution per ton of GLA is then calculated as:

$$\text{Capital cost}=\frac{\text{Cost of solketal equipment}+\text{Cost of catalyst}+\text{Cost of electrolyzer}+\text{Cost of separations equipment}}{\text{Life time of plant}\times\text{Mass of GLA produced}}$$

$$=\frac{\$2.5\times10^{6}+\$2.2\times10^{5}+\$1.33\times10^{8}+\$6.65\times10^{6}+\$1.33\times10^{7}}{10\times365\times205.26}=\$207.78\ \text{per ton of GLA}$$

(5) Operation cost

The operation cost is set to 10% of the capital costs. Accordingly, the operation cost is calculated as：

$$\text{Operation cost}=\$207.78\times0.1=\$20.78\ \text{per ton of GLA}$$

(6) Maintenance cost

The maintenance cost is assumed to be 10% of the capital cost. Accordingly, the maintenance cost is calculated as:

$$\text{Maintenance cost}=\$207.78\times0.1=\$20.78\ \text{per ton of GLA}$$

(7) Balance-of-plant cost

The balance-of-plant cost is assumed to be 58% of the capital cost and calculated as:

$$\text{Balance of plant cost}=\$207.78\times0.58=\$120.51\ \text{per ton of GLA}$$

The total cost:

$$\text{Total cost}=\$2922.11+\$217.78+\$1409.53+\$207.78+\$20.78\times2+\$120.51$$

$$=\$4919.27 \text{ per ton of GLA}$$

Potential profit:

The daily profit can be calculated based on the market price of GLA:

$$\text{Profit per ton GLA}=\$7500-\$4919.27=\$2580.73 \text{ per ton}$$

Given a daily production of 205.26 tons of GLA, the daily profit from GLA is:

$$\text{GLA profit per day}=\$2580.73\times205.26=\$529720.64$$

The Faradaic efficiency for hydrogen evolution is assumed to be 100%. The daily hydrogen production is calculated as:

$$\text{Mass of hydrogen produced per day}=\frac{\text{Q}\times\text{Molar mass of hydrogen}}{\text{N}\times\text{F}}$$

$$=\frac{9.22\times10^{11}\times2}{96485\times2}=9.55\times10^{6}\text{ g}=9.55\text{ tons}$$

Assuming a hydrogen selling price of $1900 per ton. The profit of hydrogen can be calculated as follows:

$$\text{Hydrogen profit per day}=\$1900\times9.55=\$18145$$

$$\text{Hydrogen profit per ton GLA}=\frac{\$18145}{205.26}=\$88.4$$

Assuming a K_2SO_4 selling price of $473 per ton and a daily production of 337.32 tons. The profit of K_2SO_4 can be calculated as follows:

$$K_2SO_4\text{ profit per day}=\$473\times337.32=\$159552.36$$

$$K_2SO_4\text{ profit per ton GLA}=\frac{\$159552.36}{205.26}=\$777.32$$

Assuming an acetone selling price of $603 per ton and a daily recovery of 166.7 tons. The profit of acetone can be calculated as follows:

$$\text{Acetone profit per day}=\$603\times166.7=\$100520.1$$

$$\text{Acetone profit per ton GLA}=\frac{\$100520.1}{205.26}=\$489.72$$

The total profit:

The total profit per ton of GLA, accounting for GLA production and all byproducts, is:

$$\text{Total profit}=\$2580.73+\$88.4+\$777.32+\$489.72=\$3936.17 \text{ per ton of GLA}$$

Breakeven point:

$$\text{Breakeven point}=\$7500+\$88.4+\$777.32+\$489.72=\$8855.44$$

Supplementary Tables

Table S1. EXAFS fitting parameters for $Cu_3Mo_2O_9$.

K-edge	Path	CN	R(Å)	σ^2 (10^{-3}Å^2)	ΔE_0 (eV)	R-factor
Cu	Cu–O	4.39(±1.16)	1.978	5.9(±3.73)	6.5(±1.7)	0.021
	Cu–O	1.53(±0.99)	2.308	3.4(±2.34)		
	Cu–O–Cu	3.75(±0.74)	3.020	5.7(±1.61)		
	Cu–O–Mo	4.69(±1.03)	3.614	6.6(±1.49)		
Mo	Mo–O	3.63(±0.77)	1.814	10.4(±3.10)	4.7(±2.1)	0.019
	Mo–O–Cu	5.25(±0.83)	3.549	4.8(±2.41)		

Note: The amplitude reduction factor (S_0^2), which accounts for shake-up and shake-off processes at the absorbing atom, was determined to be 1 for all samples. CN is the coordination number; R is the interatomic distance; σ^2 is the Debye-Waller factor; ΔE_0 is the edge-energy shift. The R-factor is used to evaluate the goodness of fit. All EXAFS fittings were carried out using a K-weight of 3, with fitting ranges of $3 \leqslant k$ (Å^{-1}) $\leqslant 12$ and $1.0 \leqslant R$ (Å) $\leqslant 3.8$.

Table S2. Ionic conductivity of electrolytes at different concentrations of glycerol in 1.0 M KOH.

Glycerol concentration (mM)	Ionic conductivity (μS cm^{-1})
0	195662
100	137099
200	132979
300	123251
400	116477

Table S3. Product analysis during long-term electrolysis at 1.40 V *versus* RHE during 20 cycles, each for 5 h, totaling 100 h.

Cycle number	C_{GLA}(mM)	n_{GLA}(mmol)	Conversion (%)	Selectivity (%)	Carbon Balance (%)
1	208.89	1.98	95.68	89.76	97.79
2	203.33	1.93	95.83	90.41	95.23
3	199.87	1.90	93.74	90.49	95.73
4	206.02	1.96	95.45	90.07	97.28
5	205.97	1.96	93.87	91.37	98.12
6	203.49	1.93	94.36	91.53	96.37
7	202.06	1.92	94.30	89.12	96.19
8	209.96	1.99	94.77	88.69	99.89
9	205.48	1.95	95.22	89.48	96.76
10	202.29	1.92	94.09	90.03	96.05
11	205.91	1.96	93.72	89.23	98.76
12	204.97	1.95	93.77	89.43	97.85
13	202.27	1.92	94.57	89.30	95.84
14	204.55	1.94	93.72	90.49	98.21
15	202.15	1.92	93.33	90.89	97.21
16	202.73	1.93	94.13	88.19	97.31
17	199.76	1.90	92.88	88.63	96.61
18	203.63	1.93	91.78	89.33	99.33
19	198.20	1.88	91.65	89.58	97.67
20	198.41	1.88	92.50	90.83	96.27

Note: The initial solketal concentration was 219.42 mM in a 10 mL anolyte. After each electrolysis cycle, the electrolyte volume decreased to ~9.5 mL, likely due to water evaporation and electro-osmotic drag.

Table S4. Metal concentrations (mg L^{-1}) in the electrolyte before and after long-term testing, as determined by ICP-MS analysis.

	Cu	Mo
Before reaction	0.0010	0.0104
After SOR	0.0345	0.0652
After GOR	0.1021	0.1913

Table S5. Comparison of the glycerol-to-GLA electrooxidation performances reported in the literature and in this work. ("-" denotes that the corresponding parameter was not reported or could not be extracted from the referenced study).

Electrocatalyst	Electrolyte	Reaction conditions	GLA selectivity (%)	Glycerol conversion rate (μmol h^{-1})	Glycerol conversion (%)	Reference
$Cu_3Mo_2O_9$/NF	6.0 M KOH+0.2 M Solketal	1.4 V *versus* RHE, Room temperature	90.1	695.21	94	This work
hp-PtAu/NF	1.0 M KOH+0.5 M glycerol	0.4 V *versus* RHE, Room temperature	~33	~340	-	[S10]
$PtSb_1/TiO_x$	1.0 M KOH+0.5 M glycerol	0.4 V (Cell voltage), Room temperature	72.9	-	-	[S11]
Planar Au	0.1 M KOH+0.1 M glycerol	0.9 V *versus* RHE, Room temperature	32.56	6.51	3.72	[S12]
Au/CP	0.1 M NaOH+0.1 M glycerol	1.2 V *versus* RHE, Room temperature	40	90	30	[S13]
Au-P4P/G	0.5 M NaOH+0.5 M glycerol	0.2 V *versus* HgO/Hg, Room temperature	68.6	375	7.5	[S14]
Au-CeO_2/CNT	1.0 M KOH+0.5 M glycerol	1.12 V *versus* RHE, 60℃	50.0	415.4	99.7	[S15]
Pt@G	1.0 M KOH+0.02 M glycerol	0.3-0.7 V *versus* RHE, Room temperature	81.8-74.4	-	14.7-59.4	[S16]

Pt-CeO_2/CNT	1.0 M KOH+0.1 M glycerol	0.9 V *versus* RHE, 60℃	59	213.75	85.5	[S17]
Pt/CNTs-CeO_2	1.0 M KOH+0.1 M glycerol	0.8 V *versus* RHE, 50℃	58.8	-	55.3	[S18]
Pt_5Ru_5/C	0.5 M H_2SO_4+0.1 M glycerol	1.1 V *versus* SHE, 60℃	11.1	31.5	31.5	[S19]
PtCuCoNiMn	1.0 M KOH+0.1 M glycerol	0.8 V *versus* RHE, Room temperature	75.2	-	-	[S20]
PtRhNi/GNS	1.0 M KOH+0.5 M glycerol	0.4 V *versus* SCE, Room temperature	55%	-	<5%	[S21]
Pd/CNT	4.0 M NaOH+1.0 M glycerol	0.1 V (Cell voltage) , 60℃	21.7	-	48.4	[S22]
Pd/NiCo$(OH)_2$	1.0 M KOH+0.5 M glycerol	0.72 V *versus* RHE, Room temperature	67	-	-	[S23]
Ni_3Sn	0.5 M KOH+0.5 M glycerol	1.7 V *versus* RHE, Room temperature	62	1199	-	[S24]
Co$(OH)_2$ -SDS	1.0 M KOH+0.1 M glycerol	1.42 V *versus* RHE, Room temperature	6	38.4	-	[S25]

Table S6. Prices of feedstocks and products used in the techno-economic analysis.

Feedstock/Product	Price ($/ton)	Source
	Feedstocks	
Glycerol	170	[S26]
Acetone	603	a
H_2O	0.22	[S27]
KOH	820	[S20]
H_2SO_4	45	[S27]
HCl	29.2	a
n-hexane	864	a
n-octanol	2549	a
TOMAC	5564	a
2-naphthaleneboronic acid	27384	a
	Products	
Glyceric acid	7500	[S11]
K_2SO_4	473	[S27]
Acetone	603	a
H_2	1900	[S28]

a: Market prices were obtained from ChemicalBook, Rawmex, ChemNet, and Chinese chemical market platforms. A 99% recovery rate was assumed for organic reagents.

References

[S1] G. Kresse, J. Furthmüller. Efficient iterative schemes for ab initio total-energy calculations using a plane-wave basis set. *Phys. Rev. B* **1996**, *54* (16), 11169-11186.

[S2] G. Kresse, D. Joubert. From ultrasoft pseudopotentials to the projector augmented-wave method. *Phys. Rev. B* **1999**, *59* (3), 1758-1775.

[S3] P. E. Blöchl. Projector augmented-wave method. *Phys. Rev. B* **1994**, *50* (24), 17953-17979.

[S4] J. P. Perdew, J. A. Chevary, S. H. Vosko, et al. Atoms, molecules, solids, and surfaces: Applications of the generalized gradient approximation for exchange and correlation. *Phys. Rev. B* **1992**, *46* (11), 6671-6687.

[S5] A. D. Becke. Density-functional exchange-energy approximation with correct asymptotic behavior. *Phys. Rev. A* **1988**, *38* (6), 3098-3100.

[S6] C. Lee, W. Yang, R. G. Parr. Development of the colle-salvetti correlation-energy formula into a functional of the electron density. *Phys. Rev. B* **1988**, *37* (2), 785-789.

[S7] J. P. Perdew, K. Burke, M. Ernzerhof. Generalized gradient approximation made simple. *Phys. Rev. Lett.* **1996**, *77* (18), 3865-3868.

[S8] V. L. Deringer, A. L. Tchougréeff, R. Dronskowski. Crystal orbital hamilton population (cohp) analysis as projected from plane-wave basis sets. *J. Phys. Chem. A* **2011**, *115* (21), 5461-5466.

[S9] S. Maintz, V. L. Deringer, A. L. Tchougréeff, R. Dronskowski. Lobster: A tool to extract chemical bonding from plane-wave based DFT. *J. Comput. Chem.* **2016**, *37* (11), 1030-1035.

[S10] Y. Li, X. Wei, R. Pan, et al. PtAu alloying-modulated hydroxyl and substrate adsorption for glycerol electrooxidation to C_3 products. *Energy Environ. Sci.* **2024**, *17* (12), 4205-4215.

[S11] H. Chen, R. T. Gao, F. Yang, L. Wu, L. Wang. High-selectivity electrocatalytic oxidation of glycerol to C_3-glyceraldehyde coupled to H_2O_2 production. *Angew. Chem. Int. Ed.* **2025**, *64* (48), e202514737.

[S12] D. Kim, L. S. Oh, Y. C. Tan, H. Song, H. J. Kim, J. Oh. Enhancing glycerol conversion and selectivity toward glycolic acid via precise nanostructuring of electrocatalysts. *ACS Catal.* **2021**, *11* (24), 14926-14931.

[S13] Y. Xie, L. Sun, X. Pan, et al. Carbon paper supported gold nanoflowers for tunable glycerol electrooxidation boosting efficient hydrogen evolution. *Carbon.* **2023**, *203*, 88-96.

[S14] H. Wang, L. Thia, N. Li, X. Ge, Z. Liu, X. Wang. Selective electro-oxidation of glycerol over Au supported on extended poly(4-vinylpyridine) functionalized graphene. *Appl. Catal. B.* **2015**, *166-167*, 25-31.

[S15] T. Chen, C. Guan, F. Yao, et al. Electrosynthesis of glyceric acid from glycerol oxidation on Au–CeO_2/CNT catalyst. *J. Rare Earths.* **2025**, *43* (3), 453-461.

[S16] W. Chen, L. Zhang, L. Xu, et al. Pulse potential mediated selectivity for the electrocatalytic oxidation of glycerol to glyceric acid. *Nat. Commun.* **2024**, *15* (1), 2420.

[S17] J. Li, Z. Li, Z. Zheng, et al. Tuning the product selectivity toward the high yield of glyceric acid in Pt−CeO_2/CNT electrocatalyzed oxidation of glycerol. *ChemCatChem.* **2022**, *14* (16), e202200509.

[S18] X. Liu, C. Yang. Electrocatalytic selective oxidation of glycerol to glyceric acid over efficient Pt/CNTs-CeO_2 catalysts. *Mater. Lett.* **2022**, *324*, 132658.

[S19] Y. Kim, H. W. Kim, S. Lee, et al. The role of ruthenium on carbon-supported ptru catalysts for electrocatalytic glycerol oxidation under acidic conditions. *ChemCatChem.* **2017**, *9* (9), 1683-1690.

[S20] S. Wang, Y. Lin, Y. Li, et al. Nanoscale high-entropy surface engineering promotes selective glycerol electro-oxidation to glycerate at high current density. *Nat. Nanotechnol.* **2025**, *20* (5), 646-655.

[S21] Y. Zhou, Y. Shen, J. Piao. Sustainable conversion of glycerol into value-added chemicals by selective electro-oxidation on Pt-based catalysts. *ChemElectroChem.* **2018**, *5* (13), 1636-1643.
[S22] N. Benipal, J. Qi, Q. Liu, W. Li. Carbon nanotube supported PdAg nanoparticles for electrocatalytic oxidation of glycerol in anion exchange membrane fuel cells. *Appl. Catal. B.* **2017**, *210*, 121-130.
[S23] Z. Li, N. Li, X. Zhong, et al. Enhanced hydroxyl adsorption and improved glycerol adsorption configuration for efficient glyceric acid production. *J. Colloid Interface Sci.* **2025**, *680*, 226-234.
[S24] N. Lyu, Y. Chen, A. Guan, et al. Electrocatalytic glycerol upgrading into glyceric acid on Ni_3Sn intermetallic compound. *Small.* **2024**, *20* (35), 2401872.
[S25] L. Li, Z. Zhang, H. Chen, F. Chen. Promoting electrocatalytic alcohols oxidation coupled with H_2 production via ligand intercalation strategy. *Nano Res.* **2023**, *16* (4), 4596-4602.
[S26] Y. Yan, H. Zhou, S.-M. Xu, et al. Electrocatalytic upcycling of biomass and plastic wastes to biodegradable polymer monomers and hydrogen fuel at high current densities. *J. Am. Chem. Soc.* **2023**, *145* (11), 6144-6155.
[S27] H. Zhou, Y. Ren, Z. Li, et al. Electrocatalytic upcycling of polyethylene terephthalate to commodity chemicals and H_2 fuel. *Nat. Commun.* **2021**, *12* (1), 4679.
[S28] Y. Yan, Q. Wang, J. Yang, et al. Selective electrooxidation of crude glycerol to lactic acid coupled with hydrogen production at industrially-relevant current density. *Small.* **2024**, *21* (13), 2406782.
